# Tissue-specific transcriptome profiles identify functional differences key to understanding whole plant response to life in variable salinity

**DOI:** 10.1242/bio.059147

**Published:** 2022-08-23

**Authors:** Mitchell W. Booth, Martin F. Breed, Gary A. Kendrick, Philipp E. Bayer, Anita A. Severn-Ellis, Elizabeth A. Sinclair

**Affiliations:** 1 School of Biological Sciences, The University of Western Australia, Crawley, Western Australia 6009; 2 Oceans Institute, The University of Western Australia, Crawley, Western Australia 6009, Australia; 3 College of Science and Engineering, Flinders University, Bedford Park, South Australia 5042, Australia; 4 Kings Park Science, Department of Biodiversity Conservation and Attractions, 1 Kattidj Close, West Perth, Western Australia, 6005, Australia; 5 Aquatic Animal Health Research, Indian Ocean Marine Research Centre, Department of Primary Industries and Regional Development, Watermans Bay, Western Australia, 6020, Australia

**Keywords:** Gene expression, Leaf, Meristem, *Posidonia australis*, RNA-seq, Seagrass

## Abstract

Plants endure environmental stressors via adaptation and phenotypic plasticity. Studying these mechanisms in seagrasses is extremely relevant as they are important primary producers and functionally significant carbon sinks. These mechanisms are not well understood at the tissue level in seagrasses. Using RNA-seq, we generated transcriptome sequences from tissue of leaf, basal leaf meristem and root organs of *Posidonia australis*, establishing baseline *in situ* transcriptomic profiles for tissues across a salinity gradient. Samples were collected from four *P. australis* meadows growing in Shark Bay, Western Australia. Analysis of gene expression showed significant differences between tissue types, with more variation among leaves than meristem or roots. Gene ontology enrichment analysis showed the differences were largely due to the role of photosynthesis, plant growth and nutrient absorption in leaf, meristem and root organs, respectively. Differential gene expression of leaf and meristem showed upregulation of salinity regulation processes in higher salinity meadows. Our study highlights the importance of considering leaf meristem tissue when evaluating whole-plant responses to environmental change.

This article has an associated First Person interview with the first author of the paper.

## Abstract (precis)

**Summary:** Differences in seagrass leaf, meristem and root transcriptomes across variable salinities are due to tissue-specific processes. Leaf meristem contained the broadest process range, indicating preferential use for inferring plant-wide activity.

## INTRODUCTION

The primary mechanisms that allow species to shift their phenotype in response to changing environments are adaptation and phenotypic plasticity (Hoffmann and Sgro, 2011; Williams et al., 2008). Adaptation is the progressive genetic change in populations resulting from natural selection (Bijlsma and Loeschcke, 2005). Phenotypic plasticity is the ability of a given genotype to result in a variety of different phenotypes and can be adaptive if sufficient capacity for this variety of phenotypes conveys improved fitness (Fox et al., 2019). Adaptation via plasticity can also occur through a number of mechanisms, including gene expression, epigenetics, alternative splicing and hormonal activity (Fox et al., 2019). A better understanding of the capacity of a species to respond to environmental variability through genetic adaptation or phenotypic plasticity is therefore of fundamental interest to conservation management, specifically for species at particular risk of decline due to climate change (Abelson et al., 2020; Pazzaglia et al., 2021).

Seagrasses are naturally exposed to fluctuations in biotic and abiotic stressors, such as herbivory, temperature and salinity (McDonald et al., 2016; Marín-Guirao et al., 2017; Berger et al., 2020; Heck et al., 2021). Seagrass growth, photosynthesis and nutrient allocation are among the most important processes commonly affected by these stressors (Touchette, 2007; Bell et al., 2019; Kendrick et al., 2019; Ontoria et al., 2019, 2020). How seagrasses respond to these stressors is complex and not well understood. Photosynthesis (and therefore growth) is negatively impacted under elevated temperatures, though its relationship with elevated salinity, especially in tandem with elevated temperatures, is dependent upon the plasticity or local adaptation of a given seagrass population (Malandrakis et al., 2017; Lv et al., 2018; Nguyen et al., 2021).This is demonstrated with increased nitrate assimilation in a northern hemisphere seagrass, *Zostera marina* (Lv et al., 2018). The combination of elevated salinity and temperature has shown a buffering effect of salinity on photosynthetic yield in at least one species of seagrass, *Halophila ovalis* (Ontoria et al., 2020).

The magnitude and composition of changes in seagrass gene expression will likely be tissue-dependent due to the specialisation of the different tissue types (e.g. Entrambasaguas et al., 2017). The root provides plants with a stable anchor, absorb water and nutrients and can exclude excess salt in halophytes (Flowers and Colmer, 2008; Hodge et al., 2009). Leaves harvest light and carbon dioxide (CO_2_) to produce sugar and oxygen (Barton, 2010). Gas exchange is controlled by a porous cuticle with a lacunae network, as seagrasses do not have stomata (Hemminga and Duarte, 2000). No stomatal genes have been found across two seagrass lineages: Zosteraceae (*Zostera marina*, *Zostera muelleri*) and Hydrocharitaceae (*Halophila ovalis*) (Golicz et al., 2015; Olsen et al., 2016; Lee et al., 2018).

Seagrass transcriptomic data, to date, has been predominately collected from mature leaves, despite reports that the leaf age can directly influence transcriptomic activity (Ruocco et al., 2019). Leaf sampling based on length and age may therefore result in unintended comparisons of leaves of different ages. Alternative or additional organ types may instead give more comparable snapshots of gene expression profiles across populations and environmental gradients (Pernice et al., 2016; Lin et al., 2018). Multiple seagrass tissues have been investigated via differential gene expression (Kakinuma et al., 2012; Greco et al., 2012; Schliep et al., 2015; Pernice et al., 2016; Shivaraj et al., 2017; Entrambasaguas et al., 2017; Gamain et al., 2018; Lin et al., 2018; Ruocco et al., 2020; Pazzaglia et al., 2022), with four specifically analysing leaf meristems (Greco et al., 2012; Shivaraj et al., 2017; Ruocco et al., 2020; Pazzaglia et al., 2022). The crosstalk between organs that govern plant-wide responses to stimuli, such as leaf meristem, is poorly understood in seagrasses (Davey et al., 2016), and is therefore of particular interest for species that grow in extreme habitats, such as Shark Bay in Western Australia.

Shark Bay is a UNESCO World Heritage Site in Western Australia that offers a unique opportunity to study seagrasses under a range of environmental conditions. The range of environmental conditions are due to its location, spanning temperate and tropical zones and a horizontal salinity gradient varying from oceanic seawater at 35 practical salinity units (PSU) to >62 PSU (Walker et al., 1988). Large temperate seagrasses are the dominant primary producers and ecosystem engineers responsible for sediment stabilisation, reducing water turbidity and nutrient sequestration as well as providing rich breeding and feeding grounds for many other species (Walker et al., 1988). Seagrasses within this area experience a wide range of natural environmental conditions (Walker et al., 1988; Cambridge et al., 2017), including extreme climate events (Fraser et al., 2014), posing serious ecological and management issues. *Posidonia australis* has persisted and shown recovery through these unfavourable conditions at a faster rate than the most dominant seagrass, *Amphibolis antarctica* (Kendrick et al., 2019). Therefore, understanding the natural variability in gene expression across tissue types and environments most impacted by extreme events is crucial to understanding how this temperate seagrass can respond to natural and anthropogenically-driven environmental change.

**Fig. 1.**
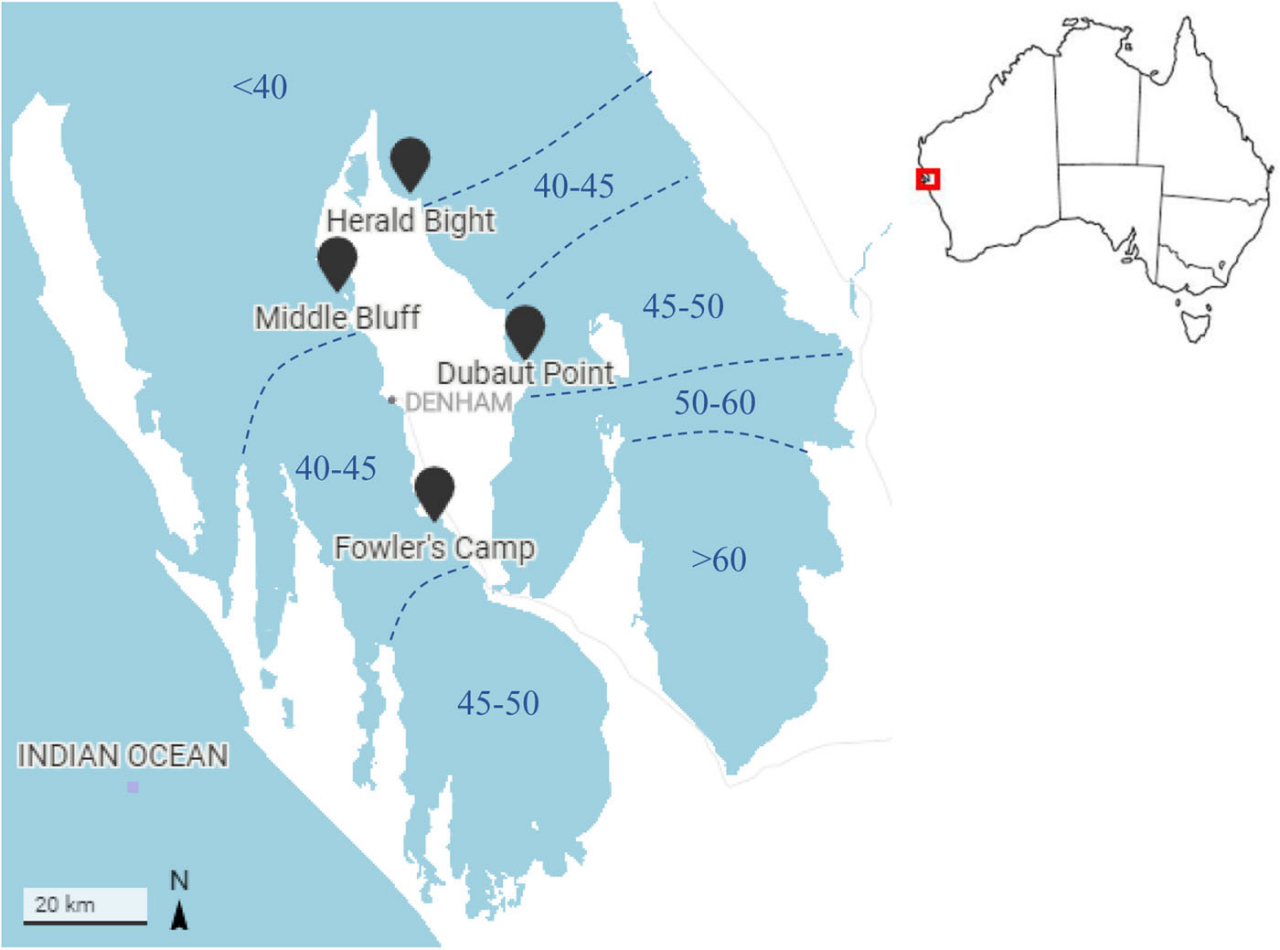
**Location of four sampled *P. australis* meadows in Shark Bay, Western Australia.** The two northern sites (Middle Bluff and Herald Bight) are lower in salinity than the southern sites (Fowlers Camp and Dubaut Point). Approximate salinity ranges in practical salinity units (PSU) are reproduced from Walker (1985).

We examined gene expression profiles in *P. australis* from four natural meadows, two each of higher and lower salinity sites within Shark Bay (Fig. 1). Gene expression data were generated for tissue samples from leaf, meristem and root organs to capture key processes for survival in the metahaline environment, which was most impacted by a marine heatwave (Strydom et al., 2020). We addressed the following questions: (1) how differentiated are gene expression profiles between tissues? (2) Are the functional differences in gene expression dependent upon tissue type? and (3) How do gene expression profiles within tissues vary between samples from different salinities?

Our transcriptome dataset describes *P. australis* growing within its natural range across a salinity gradient and will provide a resource for possible stress events in the future. These data provide a new comparative genomic resource for understanding marine plant responses in the naturally variable environment of Shark Bay.

## RESULTS

### Transcriptome sequencing and assembly

Extracted RNA was sequenced at a depth of 20 million reads, resulting in a total of 1,551,303,078 raw 150 bp paired-end reads across all samples (Table S1). FastQC analysis showed that all samples contained mean Phred scores >30 (Table S1), indicating these were high quality reads with no need for trimming and filtering due to redundancy in downstream programs. On average, 85.8% of reads for each sample were mapped to the unpublished *P. australis* genome (Table S1), resulting in 81,886 non-redundant transcripts with a mean length of 2113 bp. Briefly the assembly statistics of the *P. australis* genome used in this study contained a total size of 1215 Mbp, N50 score of 9415 and 258,843 contigs longer than 1kbp (Philipp Bayer and others, unpublished data).

### Differential gene expression

**Fig. 2.**
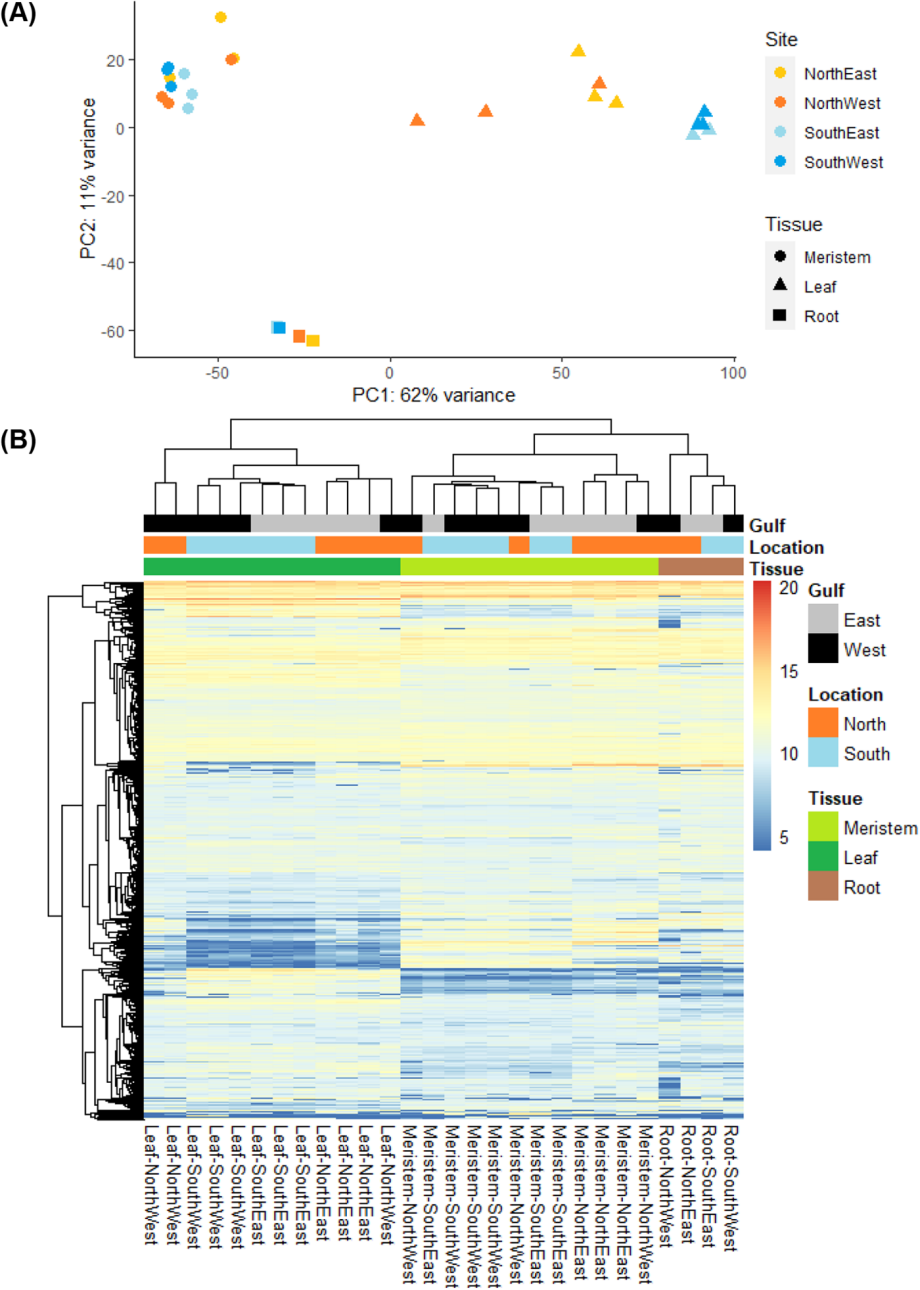
**Gene expression variance among leaf, meristem and root samples.** (A) Principal components analysis of all transcripts from *P. australis* biological samples; leaf (*n*=12), meristem (*n*=12) and root (*n*=4). (B) Heatmap of all variance-stabilising transformed counts for the top 10,000 transcripts. Organ type and derived sampling sites are represented according to the legends shown.

Gene expression profiles of *P. australis* leaf, meristem and root tissue from four sites were distinct, with organ types clustering separately (Fig. 2A,B). Both meristem and roots showed distinct separation from leaf tissue along PC1, while meristem and leaf were more distinct from root tissues along PC2 (Fig. 2A). The models were shown to be a good fit for the data, with no significantly anomalous samples for any tissue found from an outlier investigation (Figs S1–S3). Transcript count values from all samples were deemed accurate and retained within the dataset. There was more variation among leaf samples, particularly in the north-western samples, than among meristem and root samples (Fig. 2A,B). The north-western root sample was distinct among root samples, as shown from the heatmap and associated dendrogram clustering (Fig. 2B). Variation among leaf samples from northern, lower salinity sites was higher than the southern, higher salinity sites, regardless of gulf (east or west). Meristem and root samples each clustered tightly along PC1 and PC2, regardless of gulf or salinity (Fig. 2A).

### Functional differences among tissue types

**Fig. 3.**
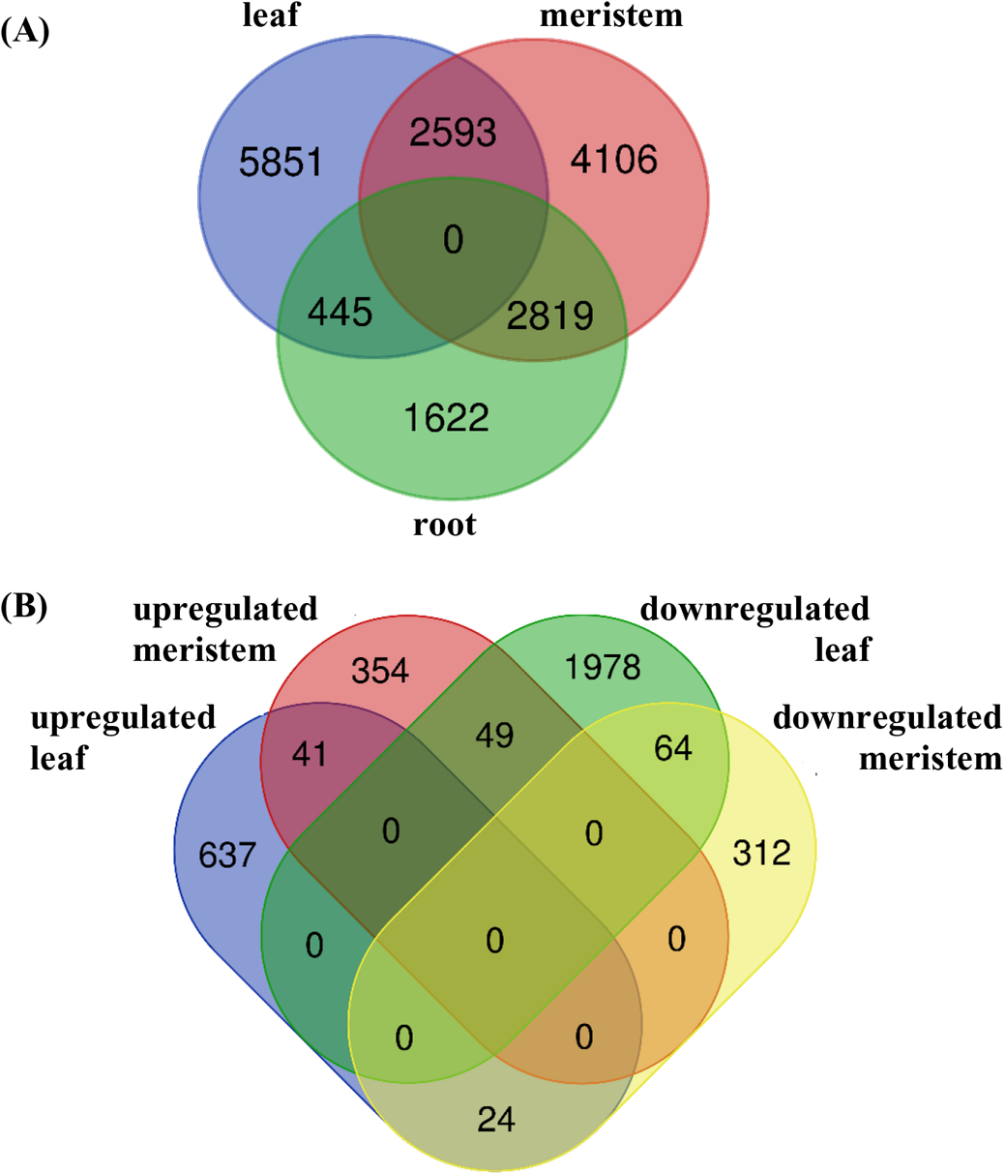
**Venn diagram of common differentially expressed genes between tissues and salinities.** Venn diagram of all significantly differentially expressed transcripts (q<0.05, LFC>|2|), found between (A) all organ type comparisons (meristem versus root, meristem versus leaf and leaf versus root), and (B) further separated by upregulated and downregulated transcripts between northern and southern meristem and leaf tissues (Upregulated meristem south versus north, downregulated meristem south versus north, upregulated leaf south versus north and downregulated leaf south versus north).

There was a low number of shared transcripts among organ tissue comparisons indicating that the majority of transcripts were unique to tissue type (Fig. 3A).

Only 1573 transcripts were shared among both groups of leaf transcripts for leaf versus meristem (3134 transcripts) and root versus leaf comparisons (5061 transcripts) (Table 1). Similarly, 349 and 978 transcripts were found shared among root and meristem comparisons, respectively.

**Table 1.**
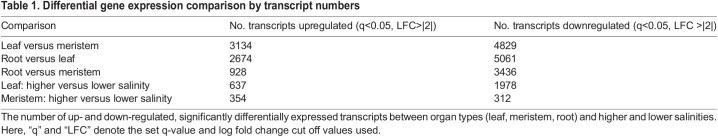
Differential gene expression comparison by transcript numbers

Leaf tissues showed a significant enrichment in processes that regulate photosynthetic development and respiration, while also exhibiting processes involved in nutrient assimilation, biosynthesis, gene expression and gene ontology (GO) terms involved in the protection from reactive oxygen species and stressors. GO terms related to core seagrass leaf processes were found commonly significantly enriched (*P*<0.05) in leaves. These processes include photosynthesis (psbS), photosystem II assembly (psbO), nitrate assimilation, vitamin E biosynthetic process (HPT, HGGT, ubiA, HST, FOLK), response to oxygen levels and circadian regulation of gene expression (Figs S4, S5, S6; Tables S2, S3). Leaves were uniquely significantly enriched relative to root tissues, for terms such as amylopectin biosynthetic process, carotenoid biosynthetic process (HST), hydrocarbon catabolic process, negative regulation of signalling, posttranscriptional gene silencing, response to abscisic acid (FOLK, DELLA), sulphate transport, zinc ion transport and response to high light intensity (psbS, psbO, FAD6, desA, ribD) (Figs S4, S5, S6; Tables S2, S3). Leaf samples with respect to meristems were significantly enriched for terms such as aerobic respiration, negative regulation of abscisic acid-activated signalling pathway, reductive pentose-phosphate cycle, selenium compound metabolic process (PAPSS, sufS), response to carbon dioxide, iron ion transport, response to iron ion and sequestering of iron ion (Figs S5, S6; Table S3). Terms such as regulation of stomatal movement, terpenoid catabolic process and stomatal movement (PHOT) were also found significantly enriched in leaves (Figs S4, S5, S6; Tables S2, S3).

Meristems were largely enriched in processes relating to plant development, such as leaf initiation and growth regulation. Meristems also showed enrichment in photosynthetic processes, terms related to stress and defence processes, as well as gene expression and regulation in either leaf or root. Significantly enriched GO terms found in meristems include microtubule depolymerisation, response to blue light (AOC), response to carbon dioxide (SUS) and sterol metabolic process (Figs S6, S7, S8; Tables S4, S5). GO terms uniquely significantly enriched with respect to the leaf tissue were axis specification, meristem maintenance, mitotic cell cycle, plant organ development, response to far-red light (AOC), sister chromatid segregation, spliceosomal snRNP assembly, cell morphogenesis, cell division and developmental growth involved in morphogenesis (Figs S6, S8; Table S5). GO terms instead uniquely significantly enriched with respect to the root were amylopectin biosynthetic process, cell fate specification, cortical microtubule organisation, leaf development, maintenance of DNA methylation, epigenetic regulation of gene expression, sulphate assimilation (PAPSS, cysC) and xylem development (Figs S6, S7; Table S4). Notably, the GO term, response to ethylene (DELLA, dapA, SQLE, ERG1, AOC), was also enriched in basal leaf meristems (Fig. S6; Table S5).

Root tissues were significantly enriched in processes regulating root growth and basic root functions. Root tissue transcripts were upregulated for GO terms involved in root growth in phosphate limited environments. The significantly enriched GO terms found commonly in root tissues were ammonium transport, phosphate ion transport (ERD6, ESL1, TM9SF2_4, XPR1, PHO1, PHO84, SLC2A13, ITR, SLC15A3_4, PHT, STP, SLC45A1_2_4), protein neddylation, response to gravity and transmembrane receptor protein serine/threonine kinase signalling pathway (Figs S6, S9, S10; Tables S6, S7). The uniquely significantly enriched terms were amine transport, callose deposition in phloem sieve plate, cellular response to hypoxia, cellular response to nutrient levels, positive gravitropism, response to water deprivation and shoot axis formation (Fig. S9; Table S6). Uniquely significantly enriched GO terms with respect to meristem were brassinosteroid biosynthetic process, cellular response to reactive nitrogen species, cinnamic acid biosynthetic process, citrate transport, lateral root formation, L-arabinose metabolic process, response to potassium ion and proximal/distal pattern formation (Fig. S10; Table S7).

### Functional differences at higher and lower salinities

**Fig. 4.**
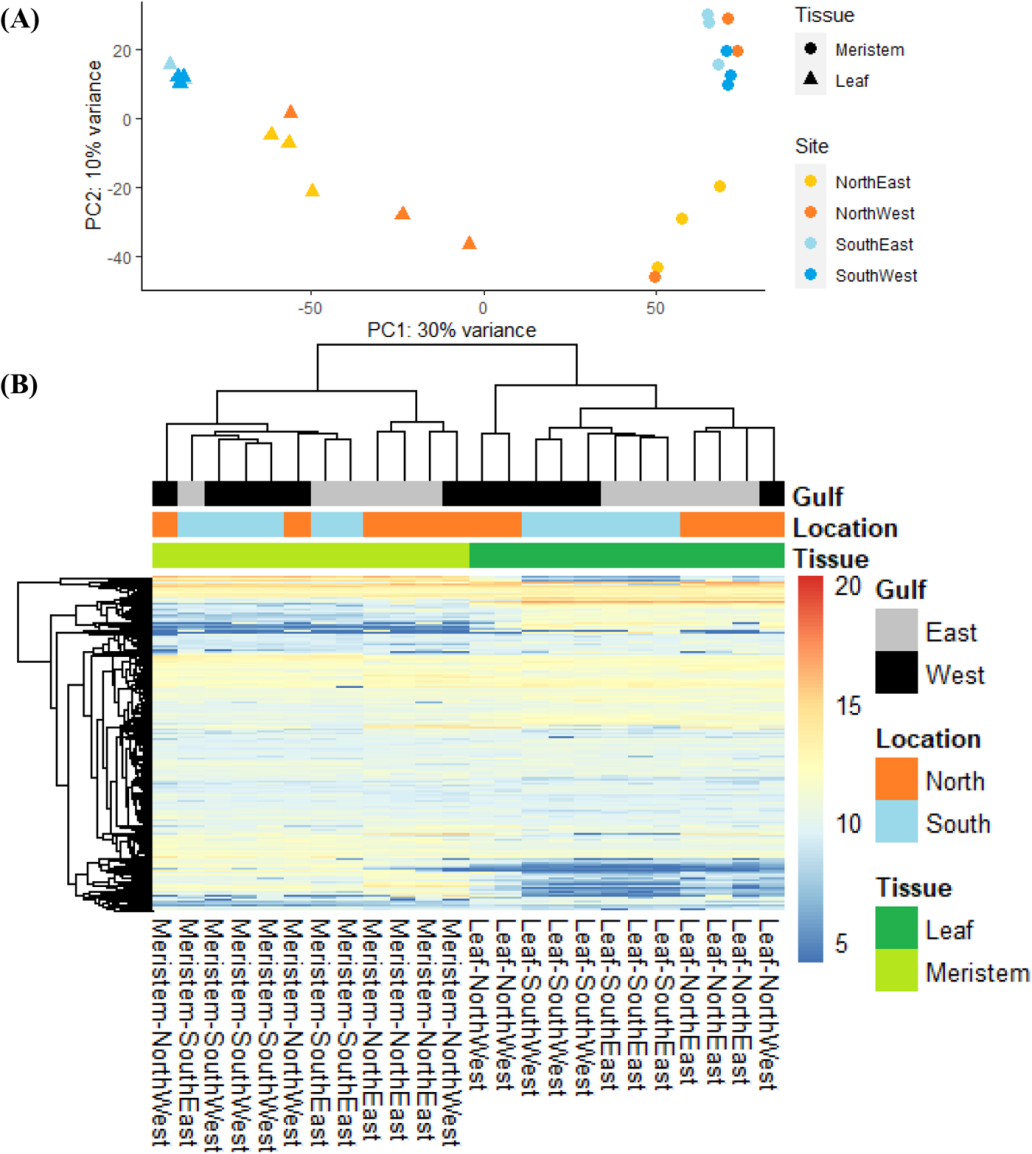
**Gene expression variance of leaf and meristem tissues from different salinities.** (A) Principal components analysis of all transcripts from *P. australis* leaf and meristem biological samples from southern, higher salinity sites (>40 PSU; Leaf *n*=6, meristem *n*=6), and northern, lower salinity sites (<40 PSU; Leaf *n*=6, meristem *n*=6). (B) Heatmap of variance-stabilising transformed counts of transcripts in leaf and meristem tissues. Tissue type and derived sampling sites are represented according to the legends shown.

The low number of shared transcripts between tissues at each salinity is consistent with the majority of transcripts being unique to tissue type. Only 41 transcripts were found commonly upregulated in leaves and meristems of higher salinity sites, and 64 transcripts were similarly upregulated in lower salinity sites (Fig. 3B). Meristems and leaves clustered independently, although leaves showed a wider spread along PC1 (Fig. 4A). Samples from the north-west were the most variable for leaf and meristems (Fig. 4A,B). All pathways involved in salinity comparisons are provided in Table 2.

**Table 2.**
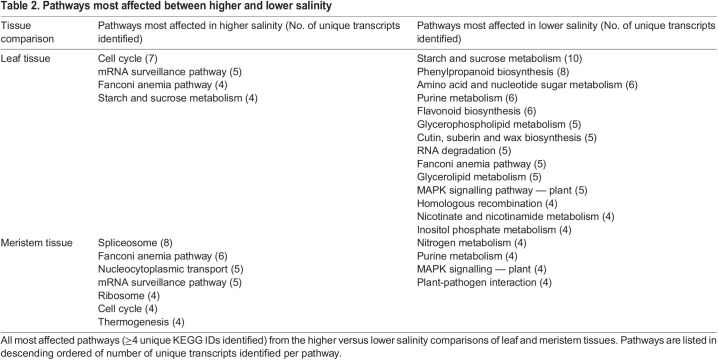
Pathways most affected between higher and lower salinity

There were no significantly enriched GO terms in common between leaf and meristem tissues from plants growing in higher salinity. However, terms such as cellular response to phosphate starvation (PLD1_2, ATP13A1, SPF1), cellular response to sucrose starvation, regulation of transcription from RNA polymerase II promoter in response to stress (INO80, INOC1) were enriched in leaf tissues (Figs S11, S12; Table S8). Pathway analysis showed transcripts from leaves in higher salinity most affect pathways involved in starch and sucrose metabolism, mRNA surveillance pathway, Fanconi anaemia pathway and cell cycle (Table 2). Meristem tissues were enriched in terms related to salinity stress and the gamma-aminobutyric acid (GABA) shunt. These terms include cellular response to salt stress (CDC2L, SPOP), defence response by callose deposition (EXOC7, EXO70), gamma-aminobutyric acid metabolic process (POP2), megasporogenesis (HORMAD, HOP1) and positive regulation of abscisic-acid-activated signalling pathway (PLD1_2) (Figs S11, S14; Table S10). The term induced systemic resistance, ethylene mediated (E3.2.1.21) in leaf tissues as well as regulation of stomatal closure (PLD1_2) in meristem tissues were also significantly enriched (Figs S11, S12, S14; Tables S8, S10). Pathways most affected contained all those found in leaves from higher salinities with the addition of thermogenesis, spliceosome, ribosome and nucleocytoplasmic transport pathways. These additional pathways may indicate that meristem tissue is more broadly affected by higher salinity than leaf tissues (Table 2).

There were no shared GO terms significantly enriched between both leaf and meristem tissues from plants growing in lower salinity. GO terms such as cellular response to osmotic stress, defence response by callose deposition (RBOH), response to hypoxia (MYBP) and response to sucrose (MYBP) were significantly enriched in leaf tissues (Figs S11, S13; Table S9). The breadth of pathways most affected reflects this broadening of enriched functional categories, with starch and sucrose metabolism, phenylpropanoid biosynthesis, amino acid and nucleotide sugar metabolism, purine metabolism and flavonoid biosynthesis pathways containing ≥6 unique KEGG IDs (Table 2). However, cell cycle and mRNA surveillance were no longer affected. Hyperosmotic salinity response (E1.11.1.7), respiratory burst involved in defence response (RBOH) and response to continuous far red-light stimulus by the high irradiance response system and regulation of stomatal movement (RBOH) were found significantly enriched in meristem tissues (Figs S11, S15; Table S11). In leaves, most pathways affected in higher salinity were also found in lower salinity. However, in meristems, none of the pathways most affected in higher salinity were found in lower salinity, as instead nitrogen metabolism, purine metabolism, MAPK signalling – plant, and plant–pathogen interaction were most affected here. These pathways were also all seen in leaves from lower salinity except nitrogen metabolism (Table 2).

## DISCUSSION

Our comparative, transcriptomic assessment from four *Posidonia australis* meadows in Shark Bay showed significant differential expression among leaf, meristem and root tissue. GO enrichment and KEGG pathway analysis showed that processes central to the role and longevity of each tissue were responsible for the differentiation of gene expression profiles at the transcript. These results were consistent with other organ-specific transcriptome studies in seagrasses (Entrambasaguas et al., 2017; Ruocco et al., 2020; Pazzaglia et al., 2022). Significant differential gene expression occurred between higher and lower salinity sites for leaf and meristem, with lower salinity sites showing more functional variation.

Significantly differentiated gene expression profiles among organ types was likely driven by organ-specific biological processes. For example, GO terms related to photosynthetic processes were most enriched in leaves, overall organ growth was most enriched in the basal leaf meristem, and sediment nutrient transport is most enriched in roots. These results support previous physiological findings from terrestrial and aquatic plants (Hemminga and Duarte, 2000; Hodge et al., 2009; Barton, 2010). GO terms associated with broad processes, such as growth and response to stimuli, were commonly enriched across all organ types, while specific terms such as ‘lateral root growth’ and ‘response to nitrate’ identified within these broad processes were highly differentiated based on the organ type. GO terms associated with responses to abiotic stimuli were also ubiquitously enriched across all tissues. However, since the seagrass samples were collected *in situ* and not recently impacted by extreme or sustained anomalous environmental stress (e.g. a marine heatwave), response to stressor stimuli terms such as ‘response to water deprivation’ in roots, likely reflects homeostatic regulatory processes, rather than regulation in response to stressor(s).

Leaves had the largest gene differentiation within organs. Similar clustering of profiles by organ types, with more variation among leaf samples than female flowers has also been observed in *Posidonia oceanica* (Entrambasaguas et al., 2017). This may be due to sampling method or small sample sizes in this study. Leaf maturity was shown to influence gene expression in *P. oceanica* using RT-qPCR (Ruocco et al., 2019), and may also explain some of the variance reported here. This was despite our best efforts to ensure samples of similar age were collected at the same time of day. Sample variance could also be explained by genotypic differences, since allelic differences may vary in gene expression (King et al., 2018 e.g. Salo et al., 2015; Procaccini et al., 2017). However, recent population genomic studies showed the four *P. australis* meadows sampled in this study belong to a single, large clone (Edgeloe et al., 2022). Therefore, wide variation in leaf samples may more likely be age-related, or possibly due to epigenetic responses associated with local environmental conditions (Richards et al., 2017).

Basal leaf meristem from *P. australis* captured a broader profile of key processes for survival in variable environments, including photosynthesis, growth, nutrient absorption and salt exclusion than either the leaf or root. We note that while transcriptomic data may infer activity of physiological or growth characteristics of *P. australis*, we do not present here either physiological or growth data, as this is currently the subject of further research. Instead, caution is advised since differential mRNA expression may not correlate to differential protein expression (Koussounadis et al., 2015). We focussed on the broad ontogenetic suite of transcripts found here and not strictly expression values suggests that meristems were more comprehensive in plant-wide activities than leaf or root. Basal leaf meristems in *P. australis* are protected within older leaf sheaths, have less contamination by (leaf) epiphytes and may be less impacted from herbivorous fish which target the leaf canopy (e.g. Bell et al., 2019). Use of meristem tissue also avoids age-dependent gene expression variation (Ruocco et al., 2019). Finally, basal leaf meristems consistently returned higher RNA quantity on a weight-by-weight basis. Therefore, we suggest that collection of the organ producing basal leaf meristem is preferable for future research to understand whole plant responses to changing environments in seagrasses.

Functional differences reported among tissues also highlighted several important pathways involved in seagrass growth and survival in extreme environments. We note that since the meadows were not experiencing any anomalous environmental stress at the time of sampling, activity in these pathways were interpreted as regular tissue function of the organs, with variation assumed to be homeostatic corrections in a naturally variable environment. Our meristem functional analysis showed enrichment of GO terms involved in both blue and far-red light. These terms were enriched due to the presence of a transcript for allene oxide cyclase (AOC). Seagrass mesocosm experiments conducted by Strydom et al., 2017 found blue light negatively impacted *Halophila ovalis* growth, possibly due to the high energy wavelengths injuring the photosynthetic apparatus, while *Ruppia maritima* and *Halodule wrightii* showed reduced canopy branching under increased far-red light. There is evidence that *in situ P. oceanica* had distinct transcriptomic profiles in relation to depth, with shallow growing plants upregulating light harvesting pigments and photoprotection mechanisms due to higher light levels (Dattolo et al., 2014). A similar process may be occurring in *P. australis* meadows in Shark Bay as they are growing in high light intensities (Walker et al., 1988). However, it is difficult to speculate on the action of these mechanisms based on vague GO terms and the action of AOC in reference to this.

Root analyses identified a suite of phosphate ion transport genes including the phosphate transporters XPR1, PHO1, PHO84 and transmembrane 9 superfamily member 2/4, TM9SF2_4, which is over-expressed in northern sites for both root and meristems, though little is known of the function of TM9SF2_4 (Fig. S6). GO terms involved in phosphorus-limited growth identified in roots reflected the phosphorous-limitation in more southerly, higher salinity environments within Shark Bay (Fraser et al., 2012; Fourqurean et al., 2012). A likely mutualistic evolutionary relationship has formed between sediment microbes and the seagrasses, where microbes alleviate phosphorus starvation by breaking down seagrass detritus to provide higher concentrations of readily available organic phosphorus in the sediment (Fraser et al., 2012, 2018). Thus, higher concentrations of available phosphorus in sediments correlate with lower concentrations of phosphorus availability in higher saline water columns, as most free phosphorus ions are bound to minerals such as apatite and not accessible by seagrass (Paytan and McLaughlin, 2007; Fraser et al., 2012). The heavy expression of transporter genes for phosphorus in *P. australis* roots supports this hypothesis. However, we cannot state this with confidence as statistical power was low since root samples were pooled due to lower quantities of RNA.

Functional enrichment of reproductive processes was unexpectedly found enriched in roots and leaves. For example, ‘microsporogenesis’ was found enriched in roots due to a SERK1 transcript. Evidently, this process is not accurately describing the activity of the expressed SERK1 here, since SERK1 activity has been linked to the formation of lateral roots (Kwaaitaal et al., 2005). Similarly, POP2 (also known as gamma-aminobutyrate transaminase, GABA-T), which regulates GABA levels, can be involved in many developmental processes (Jalil et al., 2019). However, here it was designated as ‘pollen tube growth’ enrichment in leaves. Evidence for this relationship has been reported (Palanivelu et al., 2003), however, POP2 is more accurately described by its role in leaf development (Jalil et al., 2019). This highlights the caveat when using GO terms, as thorough exploration is required of the transcripts used to generate the enrichment analysis. Further, ethylene, stomatal and terpenoid related genes have been reportedly lost from both Zosteraceae (Golicz et al., 2015; Lee et al., 2016) and Hydrocharitaceae (Lee et al., 2018) seagrass families. However, we find ethylene, stomatal and terpenoid related biological processes were enriched in leaves and meristems, which suggests conservation of these genes within the *P. australis* genome. Stomatal processes are especially intriguing because seagrasses lost their stomata as they adapted to marine life (Kuo and den Hartog, 2007). Further assessment of these upregulated fragments may show stomatal processes and regulation were ancestral to the development of stomata in plants, or they are mislabelled or misattributed to general functions through erroneous computational inference of function from large, non-species-specific databases (Wei et al., 2020).

Gene expression profiles in *P. australis* were significantly differentiated from higher and lower salinity at the transcript level for leaf and meristem. Biological processes associated with salinity stress responses were commonly enriched across higher salinity leaf and meristem, although the transcripts identified here were highly differentiated. The wide variation in transcripts among samples in lower salinity sites may indicate a more complex interaction between salinity and other leaf factors, such as herbivory, which is known to be more prevalent in the northern meadows with lower salinities (Bell et al., 2019). Leaf tissues from higher salinity sites showed nutrient starvation in the form of sucrose and phosphate, with sucrose and starch metabolism being the most affected pathway. Meristems also showed highly affected sucrose and starch pathways, in addition to enriched processes involved in the GABA-shunt, as well as upregulation of the abscisic-acid-activated signalling pathway. GABA-shunt activity is more commonly associated with anoxic and hypoxic conditions, where recently GABA-shunt induction, namely via increases in glutamate decarboxylase, γ-aminobutyrate transaminase and succinyl semialdehyde dehydrogenase activity, has been found in wheat leaves under salt stress (Lv et al., 2018; Che-Othman et al., 2020). However, the POP2 (or GABA-T) gene is responsible for the main GABA findings here in higher salinity. The presence of GABA shunt, sucrose metabolism and amino acid processes are consistent with higher concentrations of these amino acids and sucrose activity were found in *P. australis* at higher salinities (48–54 PSU) in Shark Bay. The demand for sucrose may be indicative of sucrose as an osmolyte, as well as keeping up with the energy demands of *P. australis* at a higher salinity. Leaves and meristems from lower salinities were enriched with functions involved in a broader array of biological processes than higher salinity samples largely due to the oxidase homolog, RBOH. Additional pathway analysis supported these GO findings, with the caveat that extrapolating findings from GO terms where most are driven by a handful of genes can be misleading. Caution should be taken and further research into these findings is warranted.

This study provides novel baseline data of *in situ P. australis*. We show that processes central to the role and longevity of leaf, meristem and root at the transcript level were responsible for significant differentiation in gene expression profiles among tissue types. Few differential salinity tolerance regulatory processes were identified, with the same enriched stress responses detected in higher and lower salinity sites. These results are consistent with the recent findings that in fact the four *P. australis* meadows sampled here belong to a single plant (Edgeloe et al., 2022). Thus, our data provide an interesting perspective on how a single plant can respond to natural variation across the metahaline region in Shark Bay. The low number of differentially expressed genes found between salinities for leaf and meristem tissues is also suggestive that the enriched stress responses to salinity in higher salinity sites is likely not indicative of a true stress response to high salinity waters, but rather a homeostatic response to living in higher salinity. Our work contributes to the growing body of seagrass transcriptomic data, which is increasingly important to understanding homeostatic and stress-related gene expression profiles. Further, these data provide a good baseline for understanding salinity-related gene expression regulation via *in situ* experimental manipulation, which will guide conservation and restoration efforts under changing climates.

## MATERIALS AND METHODS

### Study location

Shark Bay, also known as Gathaagudu to the Traditional Owners, is at the interface of temperate and tropical marine ecosystems, supporting high biodiversity and many iconic marine species (Kendrick et al., 2012). It is an inverse estuary bordered by Bernier, Dorre and Dirk Hartog islands to the west. Shark Bay is divided into two gulfs by a ∼110 km long peninsula and has a horizontal salinity gradient from oceanic to hypersaline water (∼35 – >70 PSU; Fig. 1). The salinity gradient has been maintained since the last sea level adjustments ∼4500 years ago by the formation of *P. australis* and *Amphibolis antarctica* seagrass-dominated sills and banks that restrict water movement, ocean exchange and nutrient availability (Fraser et al., 2012; Fourqurean et al., 2012; Kendrick et al., 2012). The gradient is steepest in the eastern gulf, where the physiological limits for seagrasses occur at the Faure Sill, the northern boundary of the hypersaline Hamelin Pool.

### Plant sampling

Four established *P. australis* meadows were sampled within the metahaline region (∼37–45 PSU) of Shark Bay, Western Australia in August 2018: northern sites, Herald Bight (25.62208° S 113.59095° E) and Middle Bluff (25.82398° S 113.46401° E) with lower salinity (<40 PSU) and southern sites, Fowlers Camp (26.10549° S 113.61285° E) and Dubaut Point (25.85282° S 113.76023° E) with higher salinity (>40 PSU; Fig. 1). The northern and southern sites within each gulf occurred are approximately 60 km apart with non-overlapping salinities. The seagrass meadows were dominated by *P. australis*, at a maximum depth of 1–2 m, away from tidal channels were selected at each location. Samples from each gulf were collected over two consecutive days between 10 am and 11 am to minimise differences in gene expression profiles due to daily plant activity cycles (de Montaigu et al., 2015; Ruocco et al., 2021). Three rhizomes containing a growing tip with three to four shoots each were collected on SCUBA from the edge of each meadow at a minimum distance of 2 m between collections. Samples were harvested in individual calico bags and transported to the research vessel for immediate processing. Approximately 2 cm of tissue was collected for each basal leaf meristem, central mature leaf tissue and root tissue (from rhizome towards the growing tip). We note that the basal leaf meristem comprised young tissue with initial and actively dividing cells where cell differentiation and post-embryonic growth occurs (Barton, 2010). For the purpose of this paper, basal leaf meristems are represented as an organ, although leaf, meristem or roots in reference to the study samples denote the tissue collected and should not be considered as the entirety of their respective organs unless otherwise stated. Samples were manually cleaned of epiphytes, as necessary, before placing in pre-labelled 2 ml screw-cap tubes and stored in a dry shipper prechilled with liquid nitrogen. All samples were processed within 10 min of harvesting to limit RNA degradation. Samples were stored at −80°C upon return to Perth, prior to RNA extraction.

### RNA extraction and library preparation

50–100 mg of plant tissue from each of the three tissues (leaf, meristem and root) was weighed separately from each sample (*n*=3) and placed into a 2 ml screw-cap tube with six 3 mm spherical YSZ grinding media beads. Samples were placed in prechilled (−80°C) metal tube blocks, then ground into powder using the 2010 Geno/Grinder^®^ (SPEX Sample Prep, Metuchen, NJ, USA) for 1 min at 1300 rpm. RNA was then extracted using the Spectrum™ Plant Total RNA kit from Sigma-Aldrich^®^ (St Louis, MO, USA) following protocol A. Samples were assessed for RNA yield via a Qubit™ 3.0 fluorometer (Invitrogen, Carlsbad, CA, USA) and purity via a NanoDrop™ 1000 spectrophotometer (Thermo Fisher Scientific, Waltham, MA, USA). RNA was treated with the DNA-free™ Kit (Invitrogen™, Carlsbad, CA, USA). RNA quality was assessed via LabChip^®^ GX Touch™ (Perkin and Elmer, Waltham, MA, USA) 24 nucleic acid analyser where all samples' RIN values scored >7. After pooling and assessing quality, yield and purity of the root RNA, due to low individual sample yield, samples were transferred to RNA stabilisation tubes (GenTegra^®^ Pleasanton, CA, USA) and sent for poly(A) selected library preparation for mRNA sequencing by GENEWIZ^®^ (Suzhou, China).

### Transcriptome sequencing and assembly

Sequencing was carried out via Illumina^®^ Hiseq^®^ sequencing platform to generate short reads of 2×150 bp at 30× coverage. Raw fastQ sequence reads were initially assessed for quality via FastQC (http://www.bioinformatics.babraham.ac.uk/projects/fastqc/), then mapped to an unpublished *P. australis* genome assembly (unpublished data, assembled by Philipp Bayer, UWA). Mapping was done using HISAT2 v2.0.5 (http://daehwankimlab.github.io/hisat2/) (Kim et al., 2019) and samtools v1.9 (http://samtools.sourceforge.net/) (Li et al., 2009). The mapped reads were then assembled into transcripts using StringTie 2.1.1 (https://ccb.jhu.edu/software/stringtie/) (Pertea et al., 2015) and Gffcompare (https://ccb.jhu.edu/software/stringtie/gffcompare.shtml) (Pertea and Pertea, 2020) was used to compare StringTie transcripts to known transcripts. Sample files were prepared for DESeq2 by producing a transcript counts matrix using prepDE.py (http://ccb.jhu.edu/software/stringtie/index.shtml?t=manual) with Python 3.8 (https://www.python.org/downloads/release/python-380/).

### Differential gene expression

R v4.0.2 (https://www.r-project.org/) was used to examine differentiation in gene expression profiles among tissues, using variance stabilising transformation on the transcript counts from all samples. Sample clustering was visualised using the R package pheatmap v1.0.12 (https://rdrr.io/cran/pheatmap/) (Kolde, 2018) and the plotPCA function of the R package DESeq2 v1.28.0 (https://bioconductor.org/packages/release/bioc/html/DESeq2.html) (Love et al., 2014) to generate clustered heatmaps and principal component analyses, respectively.

Pairwise comparisons using differential expression analysis among tissue types (leaf, meristem and root) and salinity (north, low salinity and south, higher salinity sites) were explored with DESeq2. The model used for among tissue differences was ‘∼ Gulf+Location+Tissue’, to account for the effect of sample site by gulf (west or east) and location along the salinity gradient (north or south) and tissue type had on transcript count variation. Differences in tissue types from different salinities were modelled using ‘∼ Gulf+Group’ where the ‘Group’ field is a combination of tissue type and sampling site location along the salinity gradient (e.g. ‘LeafNorth’). An outlier investigation was undertaken by assessing a box plot of Cook's distances of all samples, as well as assessing how each model fit the data by plotting dispersion estimates for both models using the plotDispEsts function.

A results table was generated using the results function of DESeq2. Transcripts with a log_2_ fold change of two (representing a fourfold change), capturing the greater than absolute fold change alternate hypothesis with a q-value <0.05, were retained. The log_2_ fold change was then transformed using the lfcShrink function of DESeq2, specifying the ‘apeglm’ shrinkage method (Zhu et al., 2019). The subsequent list of transcripts was deemed significantly differentially expressed. Comparisons of leaf and meristem tissues along the salinity gradient were conducted. Root samples were excluded as root transcripts were pooled within site due to low biological sample numbers at each location.

### Functional annotation and pathway analysis

Functional differences in gene expression between tissues were examined using GO enrichment analysis to infer important biological processes in significantly differentially expressed transcripts. The significantly differentially expressed transcripts were examined for open reading frames, as predicted using Transdecoder v5.5.0 via the Galaxy web interface (https://github.com/TransDecoder/TransDecoder/wiki) (Afgan et al., 2018). Only the single best open reading frame (based on homology over length) per transcript was kept and translated into peptide sequences. These peptide sequences were then submitted to PANNZER2 (http://ekhidna2.biocenter.helsinki.fi/sanspanz/) (Koskinen et al., 2015) for GO term annotation. The R package TopGO v3.13 (Alexa and Rahnenfuhrer., 2016) was used to determine GO term enrichment by comparing the PANNZER2 annotation files of differentially expressed transcripts against the PANNZER2 annotation of the combined transcriptome (all raw transcripts across all samples). Only significantly enriched GO terms (*P*<0.05) were used to generate treemaps with the online tool, Revigo (http://revigo.irb.hr/) (Supek et al., 2011).

Pathway analysis was conducted for the comparisons of meristem and leaf tissues only from higher and lower salinities using the Kyoto Encyclopedia of Genes and Genomes (KEGG) pathway mapping via the KEGG Automatic Annotation Server (https://www.genome.jp/kegg/kaas/) (Moriya et al., 2007). Each list of up- or downregulated significantly differentially expressed peptide transcripts was searched using the GHOSTX parameter and assigned a KEGG Orthology Identifier using the bi-directional best hit from all available monocot species (*Oryza sativa japonica* – both RefSeq and RAPDB versions*, Aegilops tauschii, Zea mays, Phoenix dactylifera, Musa acuminata, Dendrobium catenatum, Phalaenopsis equestris and Asparagus officinalis*). Pathways were considered to be most affected if four or more unique transcripts were identified in a pathway.

## Supplementary Material

Supplementary information
