# Supplementary material for: Tissue-specific transcriptome profiles identify functional differences key to understanding whole plant response to life in variable salinity
Source: Biol Open. 2022 Aug 23;11(8):bio059147. doi: 10.1242/bio.059147 (PMC9428325; doi:10.1242/bio.059147)
Supplement: Supplementary information [file biolopen-11-059147-s1.pdf]

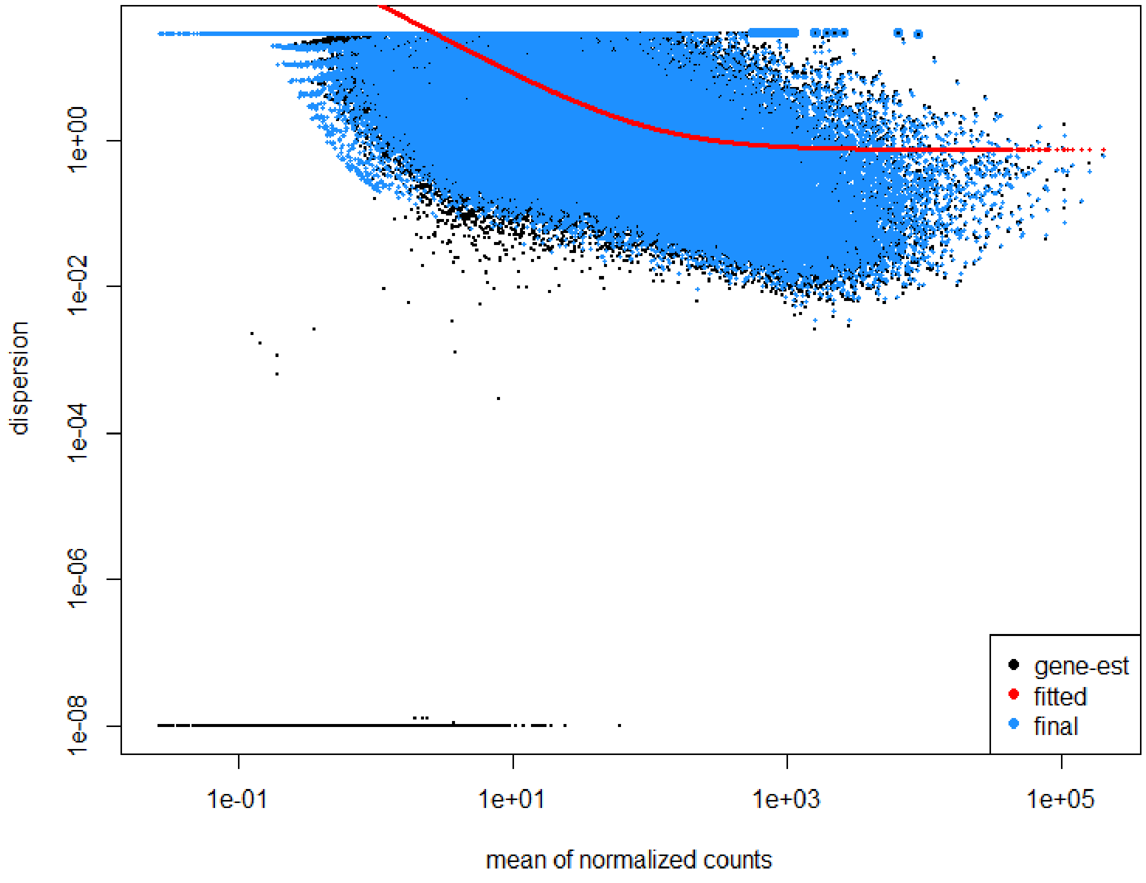

Fig. S1. Dispersion estimate plot for among sample differences.

Dispersion estimate plot of normalised count data for all transcripts across all samples for the experimental design "Gulf + Location + Tissue". Final estimates (blue) are shrunk from gene-wise estimates (black) toward the fitted estimates (red).

Biology Open • Supplementary information

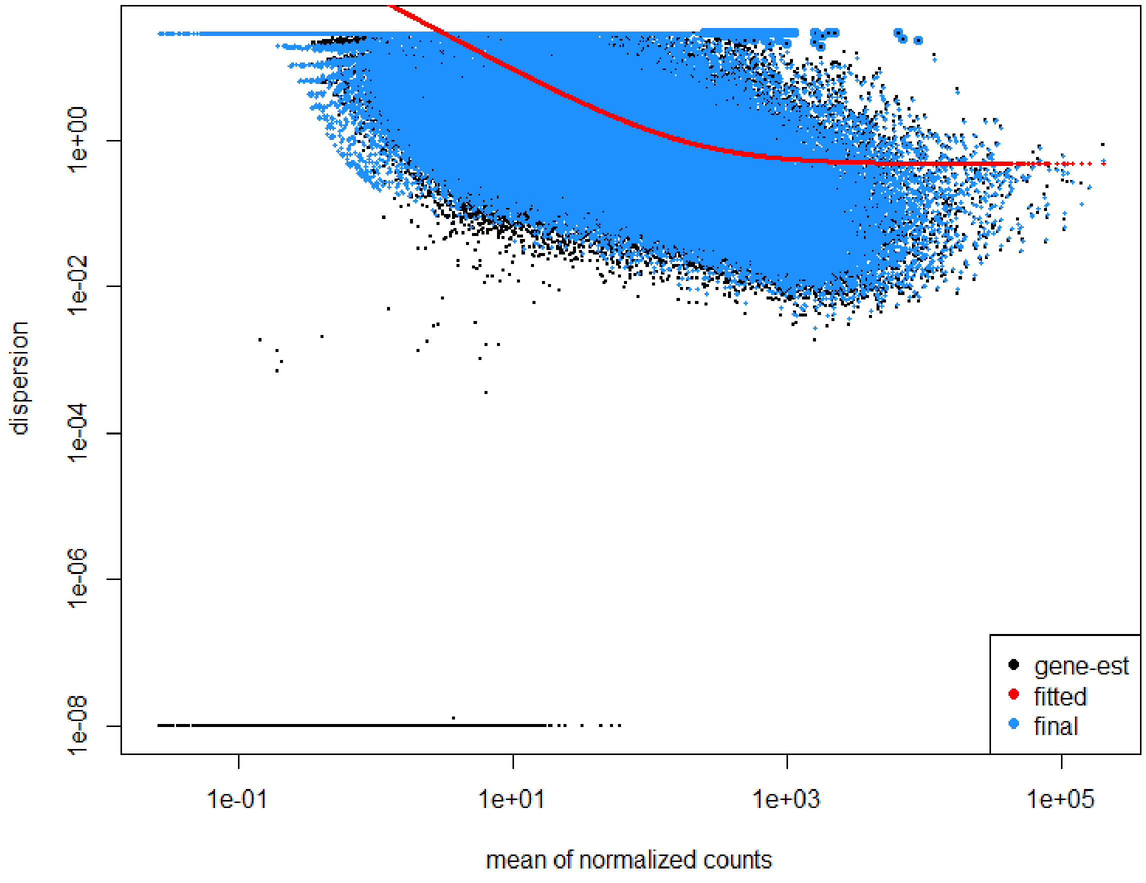

Fig. S2. Dispersion estimate plot for leaf and meristem tissues from higher and lower salinity.

Dispersion estimate plot of normalised count data for all transcripts across all leaf and meristem samples for the experimental design "Gulf + Group". Final estimates (blue) are shrunk from gene-wise estimates (black) toward the fitted estimates (red).

Biology Open • Supplementary information

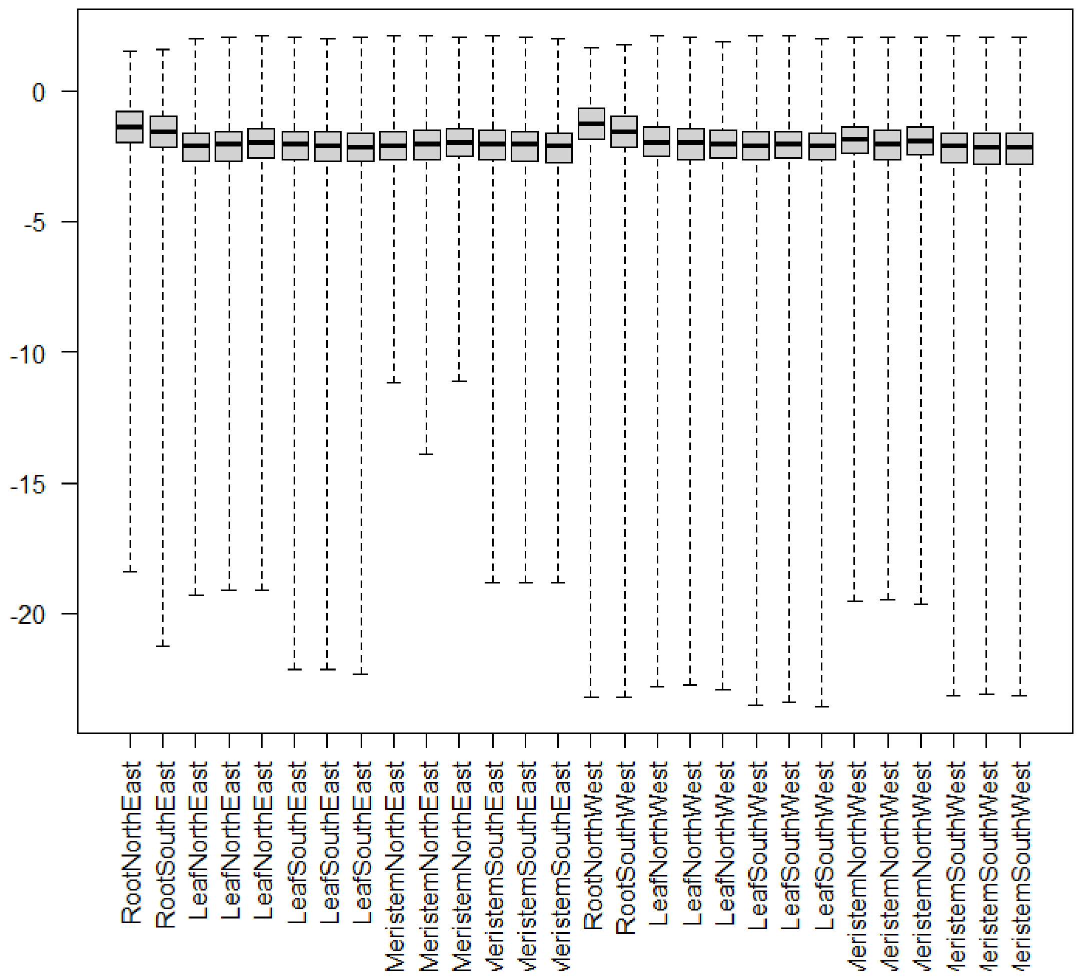

Fig. S3. Box plot of Cook's distances.

Box plot measuring how much a sample is influencing the fitted coefficients for each transcript for all samples. Cook's distances were the same for both "Gulf+Location+Tissues" and "Gulf + Group" study designs

Table S1. Transcriptome data characteristics and quality.

Number of reads, coverage, quality and reads aligned (%) to the *P. australis* genome (unpublished) for each leaf, meristem and root tissue sample.

Organ sampled	Site	Reads	Estimated coverage (1.1Gbp)	Phred score	Alignment (%)
Leaf	north-east	46,975,944	6.4	31.8	86.8
Leaf	north-east	65,100,668	8.9	31.8	87.4
Leaf	north-east	81,924,468	11.2	31.8	72.8
Leaf	north-west	48,951,734	6.7	31.5	87.4
Leaf	north-west	54,322,978	7.4	31.6	86.7
Leaf	north-west	40,092,324	5.5	31.5	86.9
Leaf	south-east	72,575,338	9.9	31.7	88.3
Leaf	south-east	66,279,626	9.0	31.7	86.1
Leaf	south-east	47,843,878	6.5	31.7	83.8
Leaf	south-west	41,077,934	5.6	31.5	87.3
Leaf	south-west	52,482,960	7.2	31.6	85.0

Biology Open • Supplementary information

Leaf	south-west	41,195,170	5.6	31.5	78.3
Meristem	north-east	56,231,086	7.7	31.8	85
Meristem	north-east	60,458,512	8.2	31.8	85.6
Meristem	north-east	61,124,298	8.3	31.8	86.9
Meristem	north-west	42,882,748	5.8	31.7	87.3
Meristem	north-west	45,440,030	6.2	31.5	85.9
Meristem	north-west	40,531,152	5.5	31.5	87.0
Meristem	south-east	58,881,470	8.0	31.8	86.7
Meristem	south-east	59,023,440	8.0	31.8	87.2
Meristem	south-east	57,265,858	7.8	31.8	86.7
Meristem	south-west	54,543,122	7.4	31.6	85.8
Meristem	south-west	64,505,128	8.8	31.6	87.2
Meristem	south-west	59,672,888	8.1	31.6	87.3
Root	north-east	56,115,670	7.7	31.6	84.7
Root	north-west	62,859,874	8.6	31.6	83.5
Root	south-east	58,338,692	8.0	31.5	88.2
Root	south-west	54,606,088	7.4	31.8	89.9

Biology Open • Supplementary information

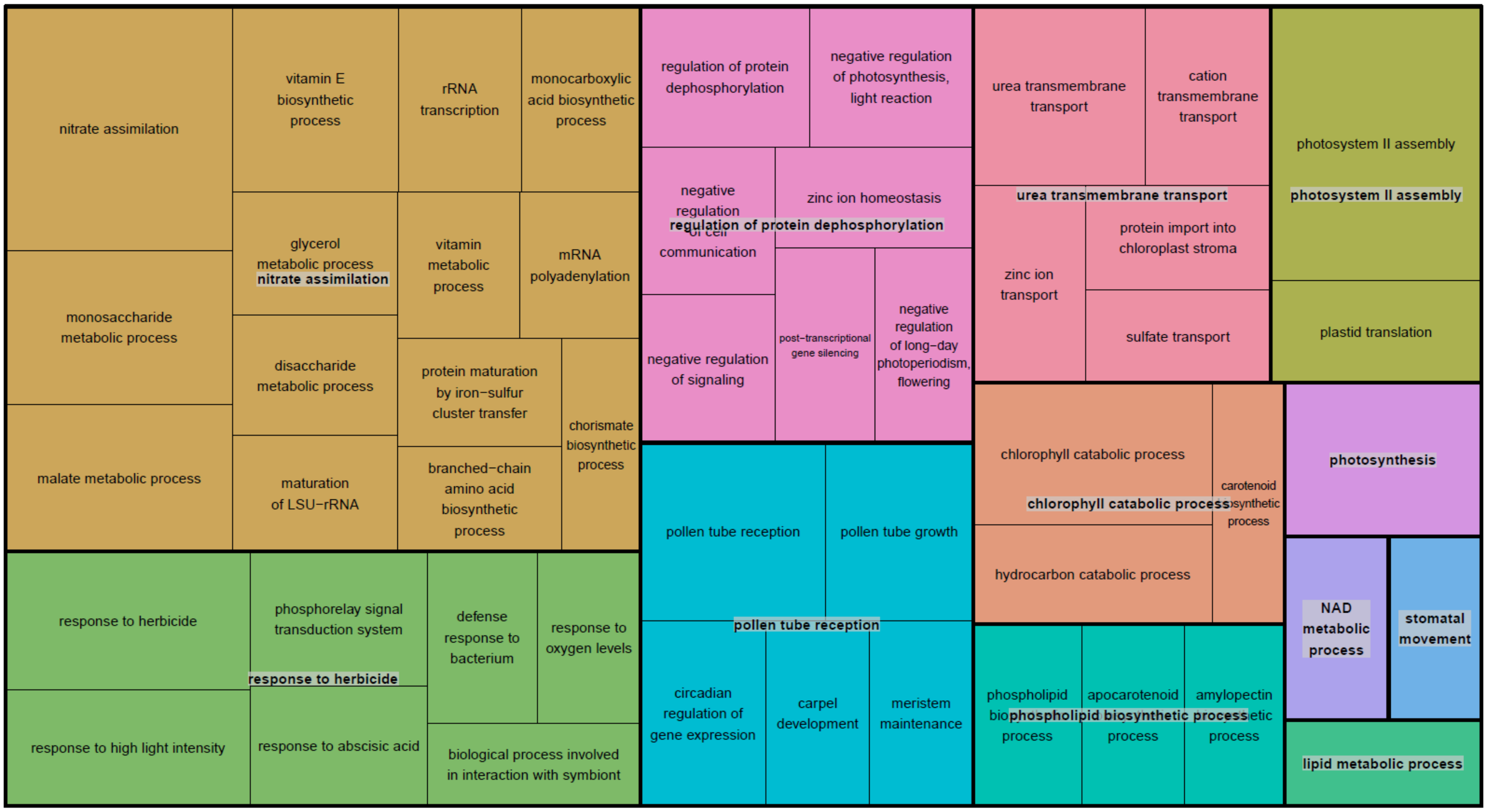

Fig. S4. GO term Treemap of leaf transcripts from root vs leaf tissue comparison.

Treemap of all significantly enriched "Biological Process" Gene Ontology terms (P<0.05) for all significantly upregulated (q<0.05, LFC>|2|) leaf transcripts from the root vs leaf organ comparison. All Gene Ontology terms were clustered into groups according to related terms to the cluster representative (group label) and coloured arbitrarily. Rectangle size reflects the absolute $\log_{10}$ value of the term's p-value.

Biology Open • Supplementary information

Table S2. Enriched GO terms of leaf transcripts from root vs leaf tissue comparison.
Table of all significantly enriched "Biological Process" Gene Ontology terms ($P<0.05$) for all significantly upregulated ($q<0.05$, $LFC>|2|$) leaf transcripts from the root vs leaf tissue comparison.

GO ID	GO Term	P-value
GO:0010207	photosystem II assembly	3.20E-05
GO:0042128	nitrate assimilation	4.90E-05
GO:0009773	photosynthetic electron transport in photosystem I	0.00023
GO:0005996	monosaccharide metabolic process	0.00194
GO:0015996	chlorophyll catabolic process	0.00227
GO:0009635	response to herbicide	0.00236
GO:0006108	malate metabolic process	0.0025
GO:0010483	pollen tube reception	0.00275
GO:0071918	urea transmembrane transport	0.00407
GO:0010189	vitamin E biosynthetic process	0.00414
GO:0015979	photosynthesis	0.0044
GO:0009644	response to high light intensity	0.00604
GO:0009860	pollen tube growth	0.00925
GO:0015995	chlorophyll biosynthetic process	0.00989
GO:0015840	urea transport	0.01421
GO:0120253	hydrocarbon catabolic process	0.01426
GO:0000160	phosphorelay signal transduction system	0.01429
GO:0035304	regulation of protein dephosphorylation	0.0143
GO:0032922	circadian regulation of gene expression	0.01569
GO:0009303	rRNA transcription	0.01653
GO:0043155	negative regulation of photosynthesis, light reaction	0.01653
GO:0098655	cation transmembrane transport	0.01801
GO:0006829	zinc ion transport	0.01883
GO:0072330	monocarboxylic acid biosynthetic process	0.01934
GO:0032544	plastid translation	0.02139
GO:0009737	response to abscisic acid	0.0229
GO:0016120	carotene biosynthetic process	0.02401
GO:0006071	glycerol metabolic process	0.02666

Biology Open • Supplementary information

GO:0005984	disaccharide metabolic process	0.0282
GO:0010648	negative regulation of cell communication	0.0282
GO:0023057	negative regulation of signaling	0.0282
GO:0055069	zinc ion homeostasis	0.0283
GO:0098660	inorganic ion transmembrane transport	0.02853
GO:0008654	phospholipid biosynthetic process	0.02888
GO:0016441	posttranscriptional gene silencing	0.03096
GO:0045037	protein import into chloroplast stroma	0.03115
GO:0048440	carpel development	0.03236
GO:0010073	meristem maintenance	0.0324
GO:0019674	NAD metabolic process	0.03252
GO:0048579	negative regulation of long-day photoperiodism, flowering	0.03262
GO:0000470	maturation of LSU-rRNA	0.03284
GO:0042742	defense response to bacterium	0.03386
GO:0043289	apocarotenoid biosynthetic process	0.03572
GO:0010021	amylopectin biosynthetic process	0.03893
GO:0006766	vitamin metabolic process	0.04133
GO:0070482	response to oxygen levels	0.04212
GO:0019405	alditol catabolic process	0.04215
GO:0051702	biological process involved in interaction with symbiont	0.04224
GO:0009687	abscisic acid metabolic process	0.04224
GO:0097428	protein maturation by iron-sulfur cluster transfer	0.04224
GO:0019288	isopentenyl diphosphate biosynthetic process, methylerythritol 4-phosphate pathway	0.04224
GO:0006378	mRNA polyadenylation	0.04224
GO:0016117	carotenoid biosynthetic process	0.04547
GO:0009082	branched-chain amino acid biosynthetic process	0.0455
GO:0008272	sulfate transport	0.04566
GO:0010118	stomatal movement	0.04747
GO:0006629	lipid metabolic process	0.04879
GO:0009423	chorismate biosynthetic process	0.04917

Biology Open • Supplementary information

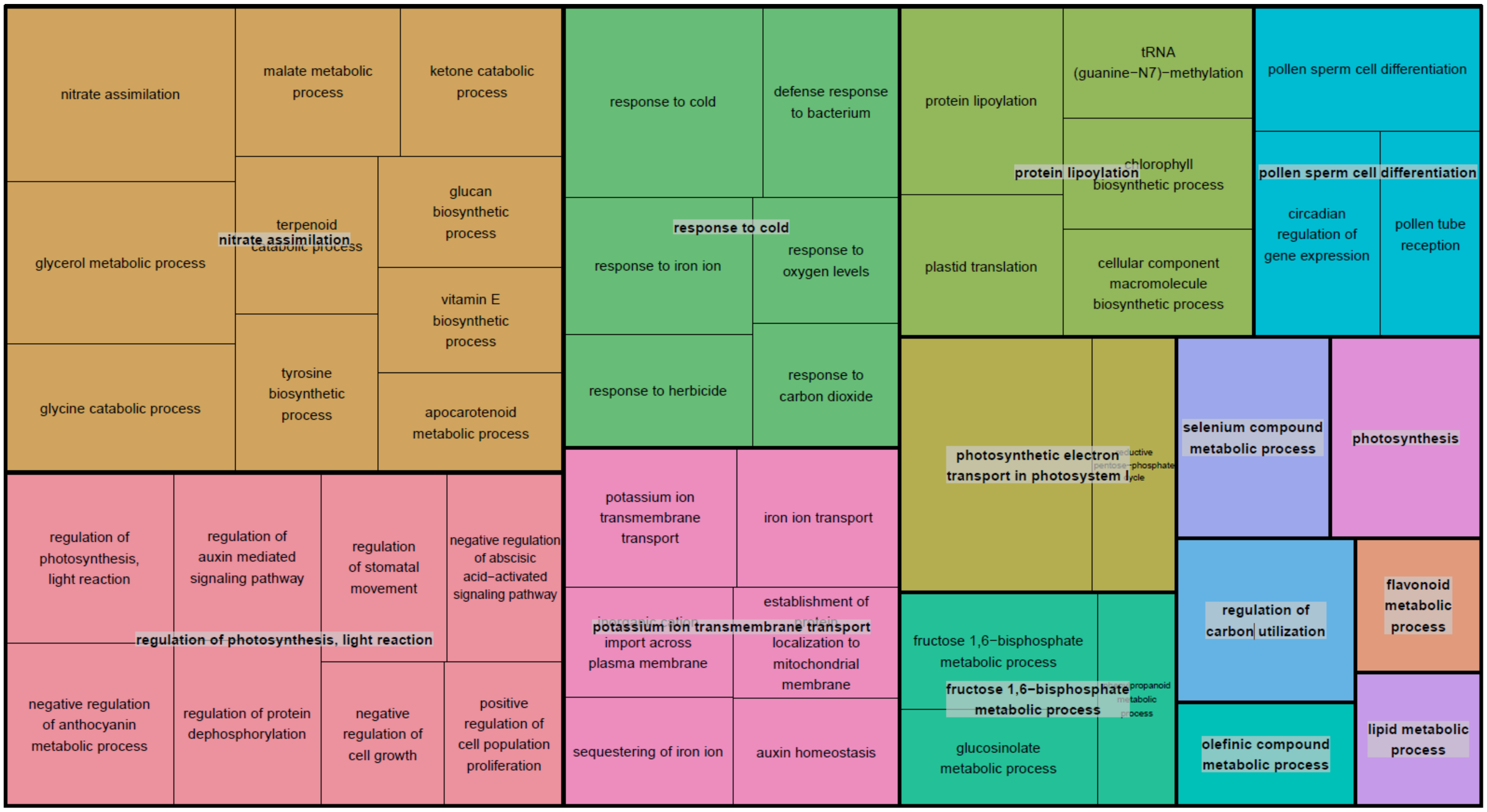

Fig. S5. GO term Treemap of leaf transcripts from leaf vs meristem tissue comparison.

Treemap of all significantly enriched “Biological Process” Gene Ontology terms (P<0.05) for all significantly upregulated (q<0.05, LFC>|2|) leaf transcripts from the leaf vs meristem tissue comparison. All Gene Ontology terms were clustered into groups according to related terms to the cluster representative (group label) and coloured arbitrarily. Rectangle size reflects the absolute $\log_{10}$ value of the term's p-value.

Biology Open • Supplementary information

Table S3. Enriched GO terms of leaf transcripts from leaf vs meristem tissue comparison.

Table of all significantly enriched “Biological Process” Gene Ontology terms (P<0.05) for all significantly upregulated (q<0.05, LFC>|2|) leaf transcripts from the leaf vs meristem tissue comparison.

GO ID	GO Term	P-value
GO:0009773	photosynthetic electron transport in photosystem I	0.00012
GO:0042128	nitrate assimilation	0.0006
GO:0009409	response to cold	0.00092
GO:0006071	glycerol metabolic process	0.00104
GO:0001887	selenium compound metabolic process	0.00344
GO:0009249	protein lipoylation	0.00347
GO:0015979	photosynthesis	0.00372
GO:0043609	regulation of carbon utilization	0.0044
GO:0006546	glycine catabolic process	0.0044
GO:0042548	regulation of photosynthesis, light reaction	0.00512
GO:0048235	pollen sperm cell differentiation	0.00545
GO:0031538	negative regulation of anthocyanin metabolic process	0.00661
GO:0046164	alcohol catabolic process	0.00779
GO:0042742	defense response to bacterium	0.00815
GO:0032922	circadian regulation of gene expression	0.00849
GO:0010039	response to iron ion	0.00849
GO:0010928	regulation of auxin mediated signaling pathway	0.01047
GO:0006108	malate metabolic process	0.01053
GO:0042182	ketone catabolic process	0.01133
GO:0035304	regulation of protein dephosphorylation	0.01134
GO:0042181	ketone biosynthetic process	0.01134
GO:0071805	potassium ion transmembrane transport	0.01156
GO:0010119	regulation of stomatal movement	0.01246
GO:0010207	photosystem II assembly	0.01282
GO:0032544	plastid translation	0.0139
GO:0030388	fructose 1,6-bisphosphate metabolic process	0.0139
GO:0006826	iron ion transport	0.01541

Biology Open • Supplementary information

GO:0016115	terpenoid catabolic process	0.01564
GO:0006571	tyrosine biosynthetic process	0.01564
GO:0009788	negative regulation of abscisic acid-activated signaling pathway	0.01743
GO:0009635	response to herbicide	0.01937
GO:0019253	reductive pentose-phosphate cycle	0.01937
GO:0106004	tRNA (guanine-N7)-methylation	0.01937
GO:0015995	chlorophyll biosynthetic process	0.02021
GO:0009765	photosynthesis, light harvesting	0.02127
GO:0010483	pollen tube reception	0.02137
GO:0009250	glucan biosynthetic process	0.02249
GO:0070589	cellular component macromolecule biosynthetic process	0.02254
GO:0007623	circadian rhythm	0.02749
GO:0010189	vitamin E biosynthetic process	0.02782
GO:0019760	glucosinolate metabolic process	0.03013
GO:0098659	inorganic cation import across plasma membrane	0.03251
GO:0070482	response to oxygen levels	0.03324
GO:0120254	olefinic compound metabolic process	0.03336
GO:0043288	apocarotenoid metabolic process	0.03352
GO:0097577	sequestering of iron ion	0.03361
GO:0019405	alditol catabolic process	0.03361
GO:0090151	establishment of protein localization to mitochondrial membrane	0.03364
GO:0010037	response to carbon dioxide	0.03496
GO:0010252	auxin homeostasis	0.03642
GO:0030308	negative regulation of cell growth	0.03748
GO:0035434	copper ion transmembrane transport	0.04006
GO:0008284	positive regulation of cell population proliferation	0.04271
GO:0098655	cation transmembrane transport	0.04427
GO:0009812	flavonoid metabolic process	0.04449
GO:0009698	phenylpropanoid metabolic process	0.04531
GO:0006629	lipid metabolic process	0.04535
GO:0009266	response to temperature stimulus	0.0475

Biology Open • Supplementary information

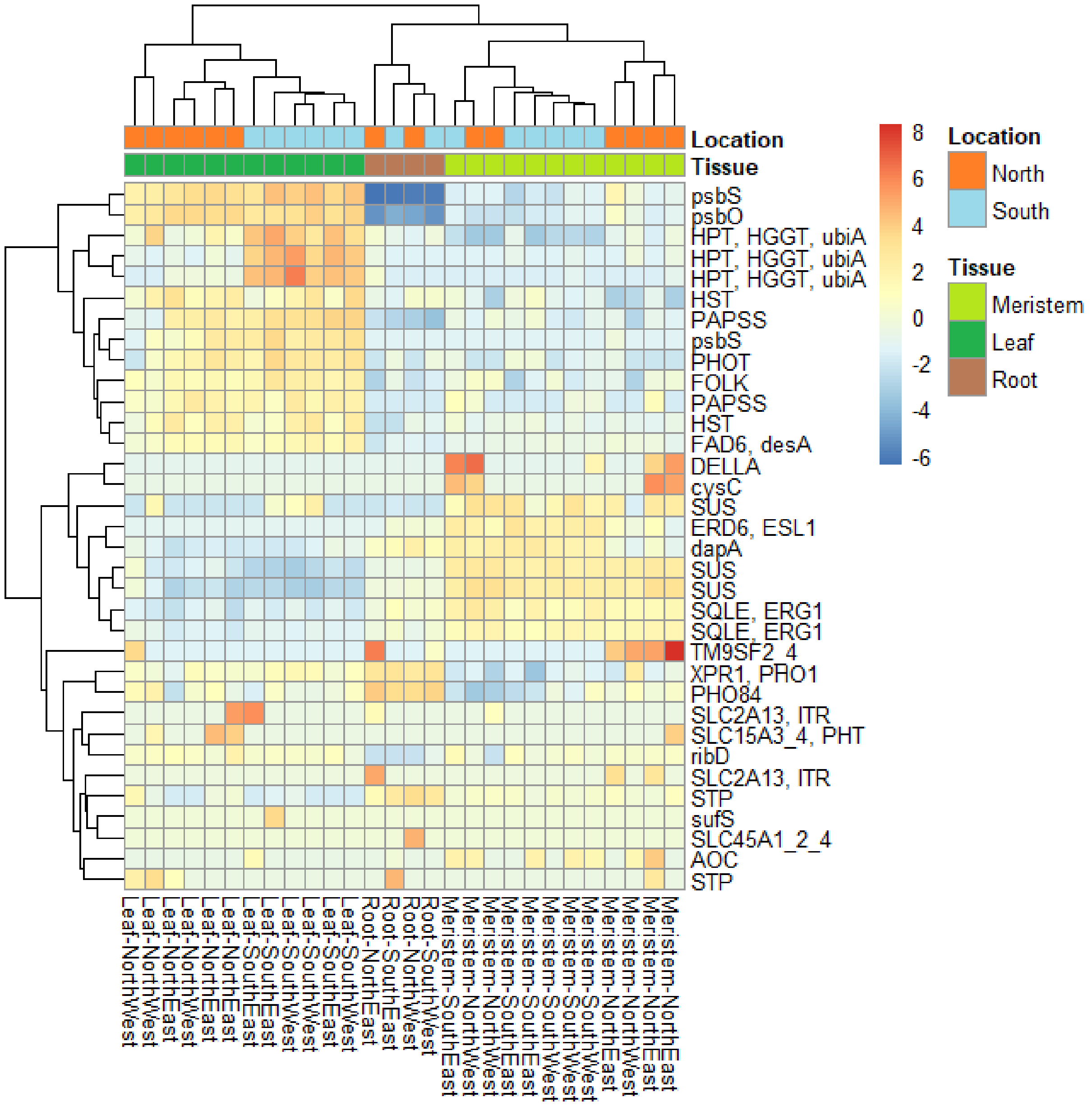

Fig. S6. Heatmap of a selection of important significantly enriched transcripts found tissue comparisons. Heatmap showing the variance stabilising transformed counts of all transcripts with KEGG annotations (shown) from a selection of important significantly enriched biological processes across all tissue types. Tissue type and derived sampling locations represented according to the legends shown.

Biology Open • Supplementary information

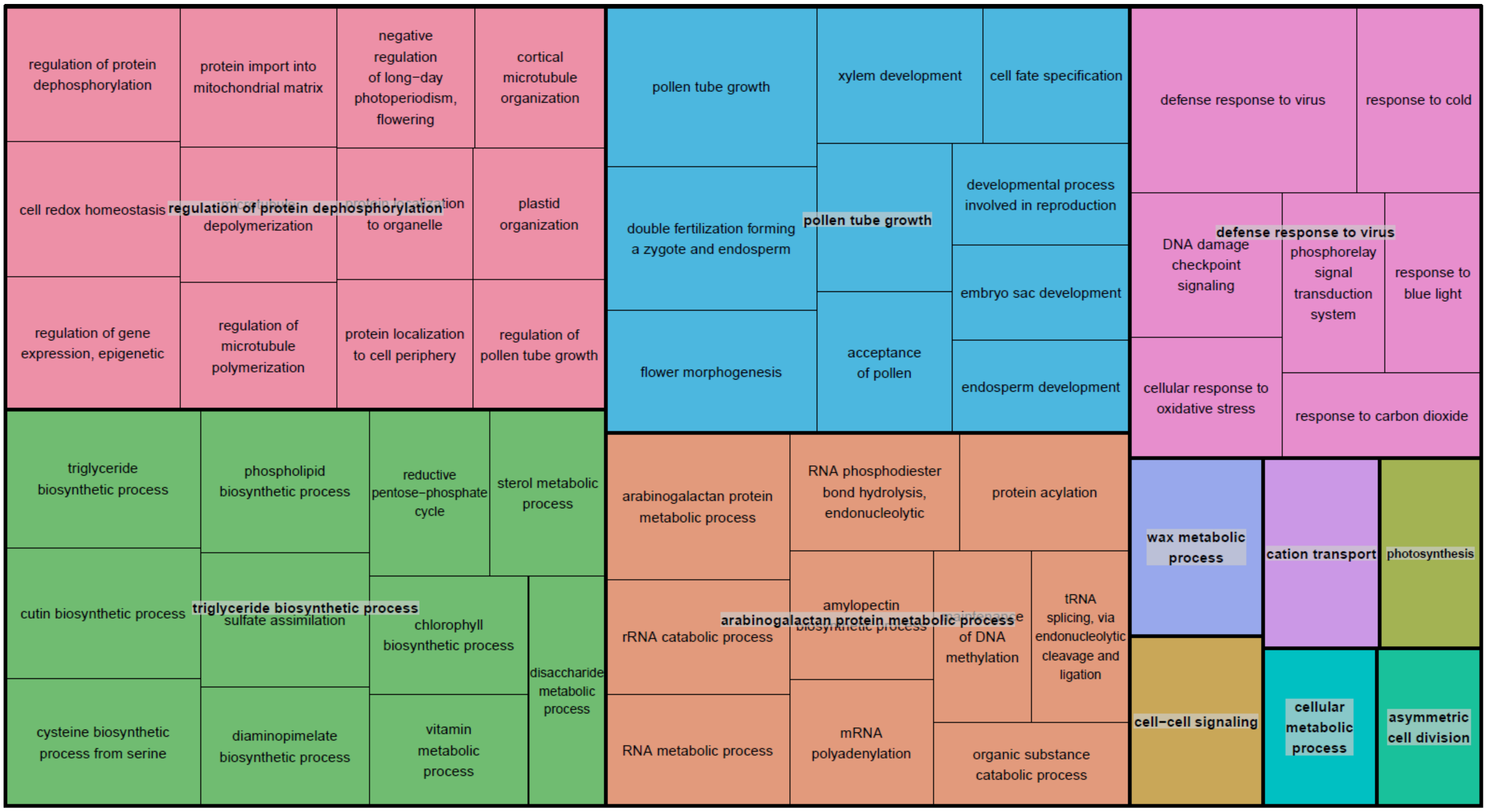

Fig. S7. GO term Treemap of meristem transcripts from root vs meristem tissue comparison.

Treemap of all significantly enriched “Biological Process” Gene Ontology terms (P<0.05) for all significantly upregulated (q<0.05, LFC>|2|) meristem transcripts from the root vs meristem tissue comparison. All Gene Ontology terms were clustered into groups according to related terms to the cluster representative (group label) and coloured arbitrarily. Rectangle size reflects the absolute $\log_{10}$ value of the term's p-value.

Biology Open • Supplementary information

Table S4. Enriched GO terms of meristem transcripts from root vs meristem tissue comparison.

Table of all significantly enriched "Biological Process" Gene Ontology terms (P<0.05) for all significantly upregulated (q<0.05, LFC>|2|) meristem transcripts from the root vs meristem tissue comparison.

GO ID	GO Term	P-value
GO:0051607	defense response to virus	0.00044
GO:0009860	pollen tube growth	0.00201
GO:0009567	double fertilization forming a zygote and endosperm	0.00386
GO:0010405	arabinogalactan protein metabolic process	0.00718
GO:0019432	triglyceride biosynthetic process	0.00718
GO:0048439	flower morphogenesis	0.0087
GO:0010143	cutin biosynthetic process	0.00909
GO:0006535	cysteine biosynthetic process from serine	0.0107
GO:0008654	phospholipid biosynthetic process	0.01212
GO:0010166	wax metabolic process	0.01302
GO:0035304	regulation of protein dephosphorylation	0.01308
GO:0045454	cell redox homeostasis	0.01348
GO:0009409	response to cold	0.01422
GO:0040029	regulation of gene expression, epigenetic	0.01426
GO:0055072	iron ion homeostasis	0.01525
GO:0010089	xylem development	0.0154
GO:0000103	sulfate assimilation	0.01605
GO:0007267	cell-cell signaling	0.01605
GO:0006812	cation transport	0.01712
GO:0000077	DNA damage checkpoint signaling	0.01748
GO:0030150	protein import into mitochondrial matrix	0.01755
GO:0007019	microtubule depolymerization	0.02044
GO:0016075	rRNA catabolic process	0.02109
GO:0016070	RNA metabolic process	0.02343
GO:0034220	ion transmembrane transport	0.02482
GO:0001708	cell fate specification	0.02498
GO:0030026	cellular manganese ion homeostasis	0.02526

Biology Open • Supplementary information

GO:0019253	reductive pentose-phosphate cycle	0.02526
GO:0019877	diaminopimelate biosynthetic process	0.02526
GO:0048366	leaf development	0.02541
GO:0090502	RNA phosphodiester bond hydrolysis, endonucleolytic	0.02577
GO:0031113	regulation of microtubule polymerization	0.02596
GO:0043543	protein acylation	0.02599
GO:0048579	negative regulation of long-day photoperiodism, flowering	0.02783
GO:0010088	phloem development	0.02783
GO:0015979	photosynthesis	0.02909
GO:0016125	sterol metabolic process	0.02954
GO:0060321	acceptance of pollen	0.0305
GO:0015995	chlorophyll biosynthetic process	0.03182
GO:0010025	wax biosynthetic process	0.03226
GO:0071427	mRNA-containing ribonucleoprotein complex export from nucleus	0.03226
GO:0043622	cortical microtubule organization	0.03326
GO:0046656	folic acid biosynthetic process	0.03326
GO:0010021	amylopectin biosynthetic process	0.03326
GO:0034599	cellular response to oxidative stress	0.03355
GO:0000160	phosphorelay signal transduction system	0.03434
GO:0006378	mRNA polyadenylation	0.03612
GO:0003006	developmental process involved in reproduction	0.03706
GO:0044237	cellular metabolic process	0.03761
GO:0033365	protein localization to organelle	0.03824
GO:0006766	vitamin metabolic process	0.03847
GO:0005984	disaccharide metabolic process	0.03854
GO:1990778	protein localization to cell periphery	0.03871
GO:0009637	response to blue light	0.0404
GO:0009657	plastid organization	0.04102
GO:0080092	regulation of pollen tube growth	0.04211
GO:0010216	maintenance of DNA methylation	0.04523
GO:0010037	response to carbon dioxide	0.04523
GO:0006388	tRNA splicing, via endonucleolytic cleavage and ligation	0.04523

Biology Open • Supplementary information

GO:0009553	embryo sac development	0.04583
GO:0080147	root hair cell development	0.04797
GO:0008356	asymmetric cell division	0.04843
GO:0009960	endosperm development	0.04843
GO:0006614	SRP-dependent cotranslational protein targeting to membrane	0.04843
GO:1901575	organic substance catabolic process	0.04998

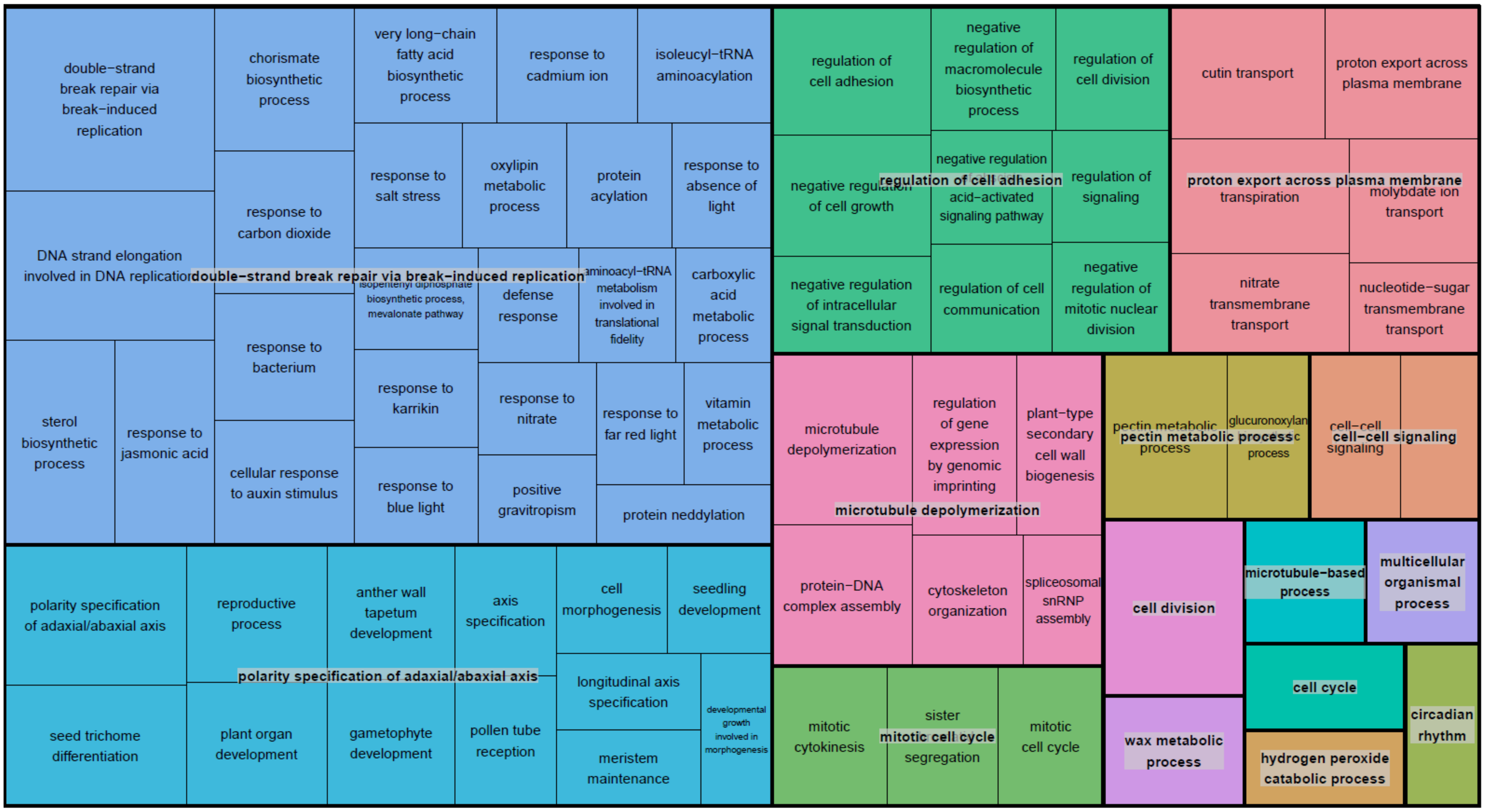

Fig. S8. GO term Treemap of meristem transcripts from leaf vs meristem tissue comparison.

Treemap of all significantly enriched "Biological Process" Gene Ontology terms ($P<0.05$) for all significantly upregulated ($q<0.05$, $LFC>|2|$) meristem transcripts from the leaf vs meristem tissue comparison. All Gene Ontology terms were clustered into groups according to related terms to the cluster representative (group label) and coloured arbitrarily. Rectangle size reflects the absolute $\log_{10}$ value of the term's p-value.

Biology Open • Supplementary information

Table S5. Enriched GO terms of meristem transcripts from leaf vs meristem tissue comparison.

Table of all significantly enriched “Biological Process” Gene Ontology terms (P<0.05) for all significantly upregulated (q<0.05, LFC>|2|) meristem transcripts from the leaf vs meristem tissue comparison.

GO ID	GO Term	P-value
GO:0000727	double-strand break repair via break-induced replication	1.50E-05
GO:0006271	DNA strand elongation involved in DNA replication	0.00013
GO:0009944	polarity specification of adaxial/abaxial axis	0.00069
GO:0051301	cell division	0.00082
GO:0007019	microtubule depolymerization	0.00101
GO:0016126	sterol biosynthetic process	0.00155
GO:0090376	seed trichome differentiation	0.0019
GO:0006270	DNA replication initiation	0.00201
GO:0045488	pectin metabolic process	0.00281
GO:0120029	proton export across plasma membrane	0.00286
GO:0010148	transpiration	0.00286
GO:0080051	cutin transport	0.00286
GO:0030155	regulation of cell adhesion	0.00286
GO:0009753	response to jasmonic acid	0.00287
GO:0009423	chorismate biosynthetic process	0.00318
GO:0065004	protein-DNA complex assembly	0.00347
GO:0010037	response to carbon dioxide	0.00356
GO:0030308	negative regulation of cell growth	0.00397
GO:0022414	reproductive process	0.00401
GO:0016125	sterol metabolic process	0.0041
GO:0010103	stomatal complex morphogenesis	0.00441
GO:0006349	regulation of gene expression by genomic imprinting	0.00441
GO:0015706	nitrate transport	0.00598
GO:0048281	inflorescence morphogenesis	0.00598
GO:0009617	response to bacterium	0.0064
GO:0009737	response to abscisic acid	0.00641
GO:0099402	plant organ development	0.00686

Biology Open • Supplementary information

GO:0071365	cellular response to auxin stimulus	0.00715
GO:0048658	anther wall tapetum development	0.00725
GO:0048439	flower morphogenesis	0.00725
GO:0009965	leaf morphogenesis	0.00739
GO:0042761	very long-chain fatty acid biosynthetic process	0.00864
GO:0046686	response to cadmium ion	0.0093
GO:0048229	gametophyte development	0.00976
GO:0000281	mitotic cytokinesis	0.01009
GO:0015689	molybdate ion transport	0.01013
GO:0009834	plant-type secondary cell wall biogenesis	0.01132
GO:0006428	isoleucyl-tRNA aminoacylation	0.01172
GO:0010166	wax metabolic process	0.01184
GO:0044042	glucan metabolic process	0.01185
GO:1902532	negative regulation of intracellular signal transduction	0.01189
GO:0000819	sister chromatid segregation	0.01191
GO:0010558	negative regulation of macromolecule biosynthetic process	0.01199
GO:0010026	trichome differentiation	0.01311
GO:0010358	leaf shaping	0.01342
GO:0007267	cell-cell signaling	0.01342
GO:0007017	microtubule-based process	0.01384
GO:0009723	response to ethylene	0.01464
GO:0000278	mitotic cell cycle	0.01497
GO:0007010	cytoskeleton organization	0.01561
GO:0051302	regulation of cell division	0.01733
GO:0032501	multicellular organismal process	0.01782
GO:0010629	negative regulation of gene expression	0.01901
GO:0010417	glucuronoxylan biosynthetic process	0.0191
GO:0007049	cell cycle	0.01914
GO:0009788	negative regulation of abscisic acid-activated signaling pathway	0.02037
GO:0009651	response to salt stress	0.02056
GO:0019877	diaminopimelate biosynthetic process	0.02118
GO:0023051	regulation of signaling	0.02293
GO:0010646	regulation of cell communication	0.02293

Biology Open • Supplementary information

GO:0009798	axis specification	0.02333
GO:0010483	pollen tube reception	0.02336
GO:0045839	negative regulation of mitotic nuclear division	0.02365
GO:0019932	second-messenger-mediated signaling	0.02365
GO:0046578	regulation of Ras protein signal transduction	0.02365
GO:0031407	oxylipin metabolic process	0.02366
GO:0009566	fertilization	0.02366
GO:0043543	protein acylation	0.02377
GO:0010025	wax biosynthetic process	0.02535
GO:0009908	flower development	0.02618
GO:0009646	response to absence of light	0.02771
GO:0019287	isopentenyl diphosphate biosynthetic process, mevalonate pathway	0.02796
GO:0042742	defense response to bacterium	0.02799
GO:0000712	resolution of meiotic recombination intermediates	0.03038
GO:0080167	response to karrikin	0.03087
GO:0042744	hydrogen peroxide catabolic process	0.03094
GO:0009637	response to blue light	0.03162
GO:0000902	cell morphogenesis	0.0321
GO:0007623	circadian rhythm	0.03231
GO:0015780	nucleotide-sugar transmembrane transport	0.03522
GO:0006952	defense response	0.03698
GO:0090351	seedling development	0.04016
GO:0009942	longitudinal axis specification	0.04086
GO:0009864	induced systemic resistance, jasmonic acid mediated signaling pathway	0.04086
GO:0010311	lateral root formation	0.04264
GO:0106074	aminoacyl-tRNA metabolism involved in translational fidelity	0.04264
GO:0019752	carboxylic acid metabolic process	0.04276
GO:0010073	meristem maintenance	0.04316
GO:0010167	response to nitrate	0.04367
GO:0060560	developmental growth involved in morphogenesis	0.04615
GO:0009958	positive gravitropism	0.04654
GO:0010218	response to far red light	0.04654

Biology Open • Supplementary information

GO:0033314	mitotic DNA replication checkpoint signaling	0.04654
GO:0006766	vitamin metabolic process	0.04705
GO:0045116	protein neddylation	0.04948
GO:0000387	spliceosomal snRNP assembly	0.04948

Biology Open • Supplementary information

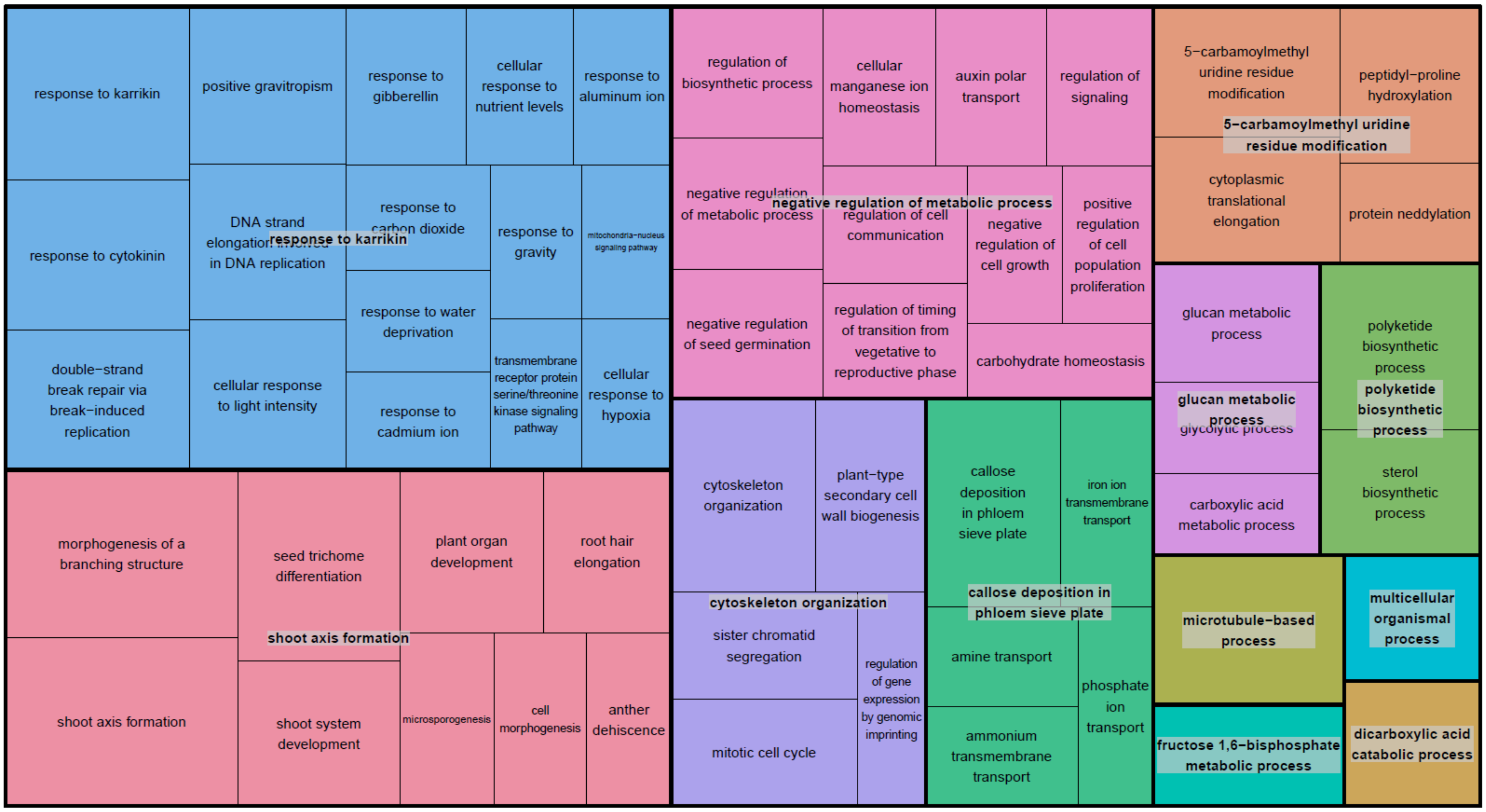

Fig. S9. **GO term Treemap of root transcripts from root vs leaf tissue comparison.**

Treemap of all significantly enriched “Biological Process” Gene Ontology terms (P<0.05) for all significantly upregulated (q<0.05, LFC>|2|) root transcripts from the root vs leaf tissue comparison. All Gene Ontology terms were clustered into groups according to related terms to the cluster representative (group label) and coloured arbitrarily. Rectangle size reflects the absolute $\log_{10}$ value of the term's p-value.

Biology Open • Supplementary information

Table S6. Enriched GO term Treemap of root transcripts from root vs leaf tissue comparison.

Table of all significantly enriched "Biological Process" Gene Ontology terms (P<0.05) for all significantly upregulated (q<0.05, LFC>|2|) root transcripts from the root vs leaf tissue comparison.

GO ID	GO Term	P-value
GO:0001763	morphogenesis of a branching structure	0.00014
GO:0010346	shoot axis formation	0.00014
GO:0080167	response to karrikin	0.00071
GO:0090376	seed trichome differentiation	0.00087
GO:0007017	microtubule-based process	0.00152
GO:0080165	callose deposition in phloem sieve plate	0.00168
GO:0007010	cytoskeleton organization	0.00175
GO:0009735	response to cytokinin	0.00186
GO:0009733	response to auxin	0.0021
GO:0030639	polyketide biosynthetic process	0.00233
GO:0000727	double-strand break repair via break-induced replication	0.00296
GO:0009958	positive gravitropism	0.00364
GO:0006271	DNA strand elongation involved in DNA replication	0.00394
GO:0080178	5-carbamoylmethyl uridine residue modification	0.00394
GO:0002182	cytoplasmic translational elongation	0.00488
GO:0071484	cellular response to light intensity	0.00488
GO:0048367	shoot system development	0.00489
GO:0099402	plant organ development	0.00491
GO:0019511	peptidyl-proline hydroxylation	0.00673
GO:0009834	plant-type secondary cell wall biogenesis	0.00794
GO:0048767	root hair elongation	0.00872
GO:0016126	sterol biosynthetic process	0.01033
GO:0009889	regulation of biosynthetic process	0.01064
GO:0009892	negative regulation of metabolic process	0.01066
GO:0000819	sister chromatid segregation	0.01069

Biology Open • Supplementary information

GO:0044042	glucan metabolic process	0.01073
GO:0000278	mitotic cell cycle	0.01091
GO:0090378	seed trichome elongation	0.01102
GO:0010187	negative regulation of seed germination	0.01173
GO:0030388	fructose 1,6-bisphosphate metabolic process	0.01248
GO:0034755	iron ion transmembrane transport	0.01248
GO:0009739	response to gibberellin	0.01338
GO:0030026	cellular manganese ion homeostasis	0.01742
GO:0009926	auxin polar transport	0.01804
GO:0009416	response to light stimulus	0.01936
GO:0032501	multicellular organismal process	0.02017
GO:0023051	regulation of signaling	0.02105
GO:0010646	regulation of cell communication	0.02106
GO:0043649	dicarboxylic acid catabolic process	0.02109
GO:0031669	cellular response to nutrient levels	0.02121
GO:0048510	regulation of timing of transition from vegetative to reproductive phase	0.02304
GO:0009556	microsporogenesis	0.02506
GO:0000902	cell morphogenesis	0.02598
GO:0010152	pollen maturation	0.02714
GO:0010044	response to aluminum ion	0.0293
GO:0010037	response to carbon dioxide	0.03152
GO:0006096	glycolytic process	0.03175
GO:0015837	amine transport	0.03177
GO:0015696	ammonium transport	0.03179
GO:0006817	phosphate ion transport	0.03272
GO:0030308	negative regulation of cell growth	0.0338
GO:0009414	response to water deprivation	0.0352
GO:0010103	stomatal complex morphogenesis	0.03615
GO:0009901	anther dehiscence	0.03615
GO:0006349	regulation of gene expression by genomic imprinting	0.03615
GO:0008284	positive regulation of cell population	0.03856

Biology Open • Supplementary information

	proliferation	
GO:0009723	response to ethylene	0.04066
GO:0046686	response to cadmium ion	0.0409
GO:0045116	protein neddylation	0.04103
GO:0060560	developmental growth involved in morphogenesis	0.04124
GO:0009629	response to gravity	0.04189
GO:0033500	carbohydrate homeostasis	0.04216
GO:0031930	mitochondria-nucleus signaling pathway	0.04355
GO:0007178	transmembrane receptor protein serine/threonine kinase signaling pathway	0.04398
GO:0071456	cellular response to hypoxia	0.04548
GO:0019752	carboxylic acid metabolic process	0.04578
GO:0071421	manganese ion transmembrane transport	0.04876

Biology Open • Supplementary information

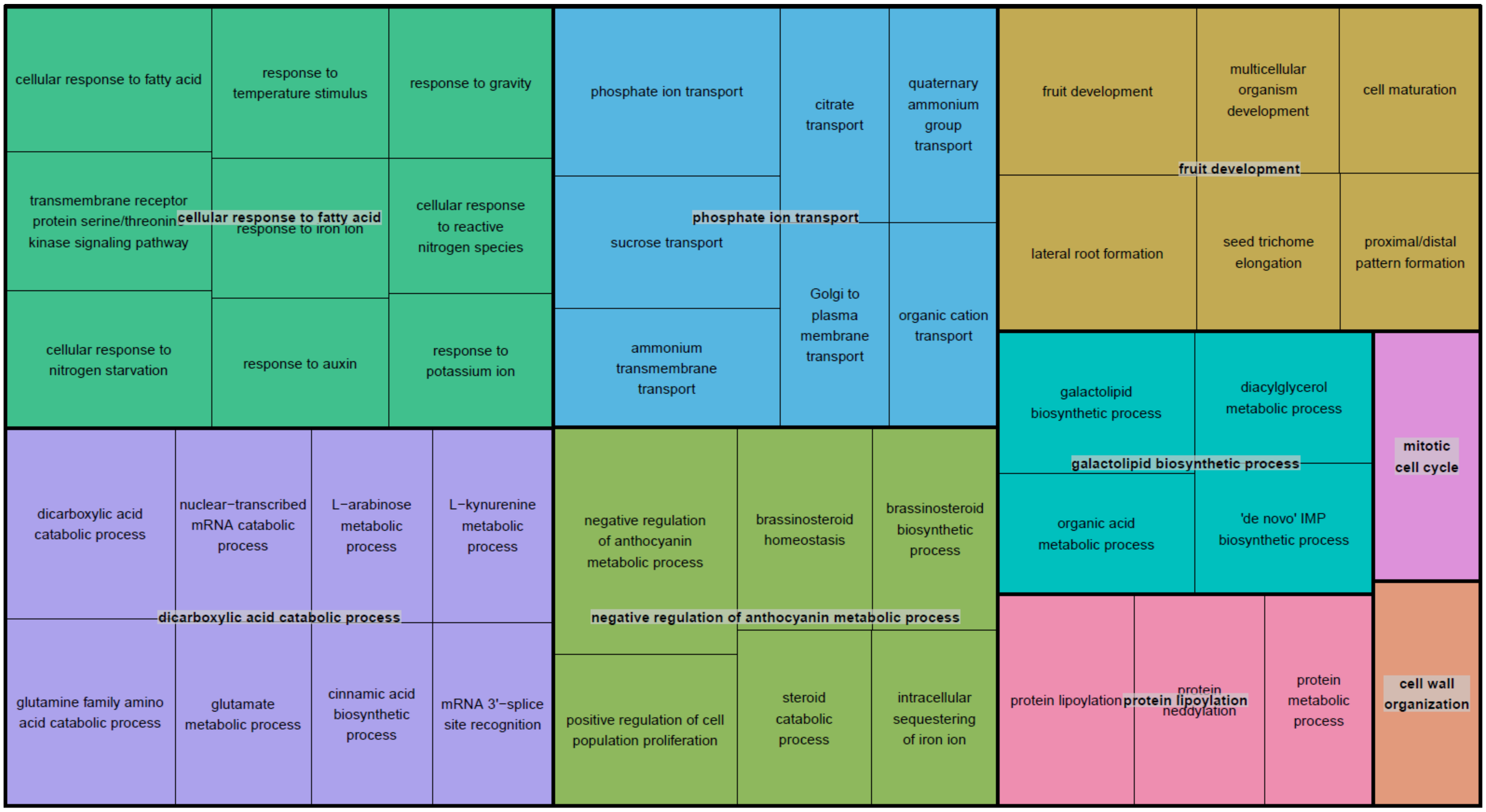

Fig. S10. GO term Treemap of root transcripts from root vs meristem tissue comparison.

Treemap of all significantly enriched “Biological Process” Gene Ontology terms (P<0.05) for all significantly upregulated (q<0.05, LFC>|2|) root transcripts from the root vs meristem tissue comparison. All Gene Ontology terms were clustered into groups according to related terms to the cluster representative (group label) and coloured arbitrarily. Rectangle size reflects the absolute $\log_{10}$ value of the term's p-value.

Biology Open • Supplementary information

Table S7. Enriched GO terms of meristem transcripts from root vs meristem tissue comparison.

Table of all significantly enriched "Biological Process" Gene Ontology terms (P<0.05) for all significantly upregulated (q<0.05, LFC>|2|) root transcripts from the root vs meristem tissue comparison.

GO ID	GO Term	P-value
GO:0031538	negative regulation of anthocyanin metabolic process	0.0029
GO:0006817	phosphate ion transport	0.0044
GO:0010154	fruit development	0.0088
GO:0043649	dicarboxylic acid catabolic process	0.0106
GO:0009065	glutamine family amino acid catabolic process	0.0116
GO:0010311	lateral root formation	0.0128
GO:0071398	cellular response to fatty acid	0.0148
GO:0015770	sucrose transport	0.0149
GO:0009249	protein lipoylation	0.0172
GO:0007178	transmembrane receptor protein serine/threonine kinase signaling pathway	0.0174
GO:0006995	cellular response to nitrogen starvation	0.0185
GO:0008284	positive regulation of cell population proliferation	0.0198
GO:0019375	galactolipid biosynthetic process	0.0198
GO:0045116	protein neddylation	0.0211
GO:0010268	brassinosteroid homeostasis	0.0211
GO:0015696	ammonium transport	0.0222
GO:0009266	response to temperature stimulus	0.0231
GO:0000278	mitotic cell cycle	0.0233
GO:0000956	nuclear-transcribed mRNA catabolic process	0.0247
GO:0016132	brassinosteroid biosynthetic process	0.0281
GO:0009629	response to gravity	0.0294
GO:0006536	glutamate metabolic process	0.0296
GO:0010039	response to iron ion	0.0312
GO:0071555	cell wall organization	0.0337
GO:0006082	organic acid metabolic process	0.0348
GO:0007275	multicellular organism development	0.0361

Biology Open • Supplementary information

GO:0048469	cell maturation	0.0366
GO:0015746	citrate transport	0.0368
GO:0006706	steroid catabolic process	0.0368
GO:0007281	germ cell development	0.0368
GO:0046373	L-arabinose metabolic process	0.0368
GO:0015697	quaternary ammonium group transport	0.0368
GO:0048530	fruit morphogenesis	0.0368
GO:0046339	diacylglycerol metabolic process	0.0368
GO:0097052	L-kynurenine metabolic process	0.0368
GO:0006189	'de novo' IMP biosynthetic process	0.0376
GO:0019538	protein metabolic process	0.0395
GO:0009733	response to auxin	0.0405
GO:0090378	seed trichome elongation	0.0411
GO:0006893	Golgi to plasma membrane transport	0.0428
GO:0006880	intracellular sequestering of iron ion	0.044
GO:0035864	response to potassium ion	0.044
GO:0009954	proximal/distal pattern formation	0.044
GO:0015695	organic cation transport	0.044
GO:0009800	cinnamic acid biosynthetic process	0.044
GO:1902170	cellular response to reactive nitrogen species	0.044
GO:0000389	mRNA 3'-splice site recognition	0.044

Biology Open • Supplementary information

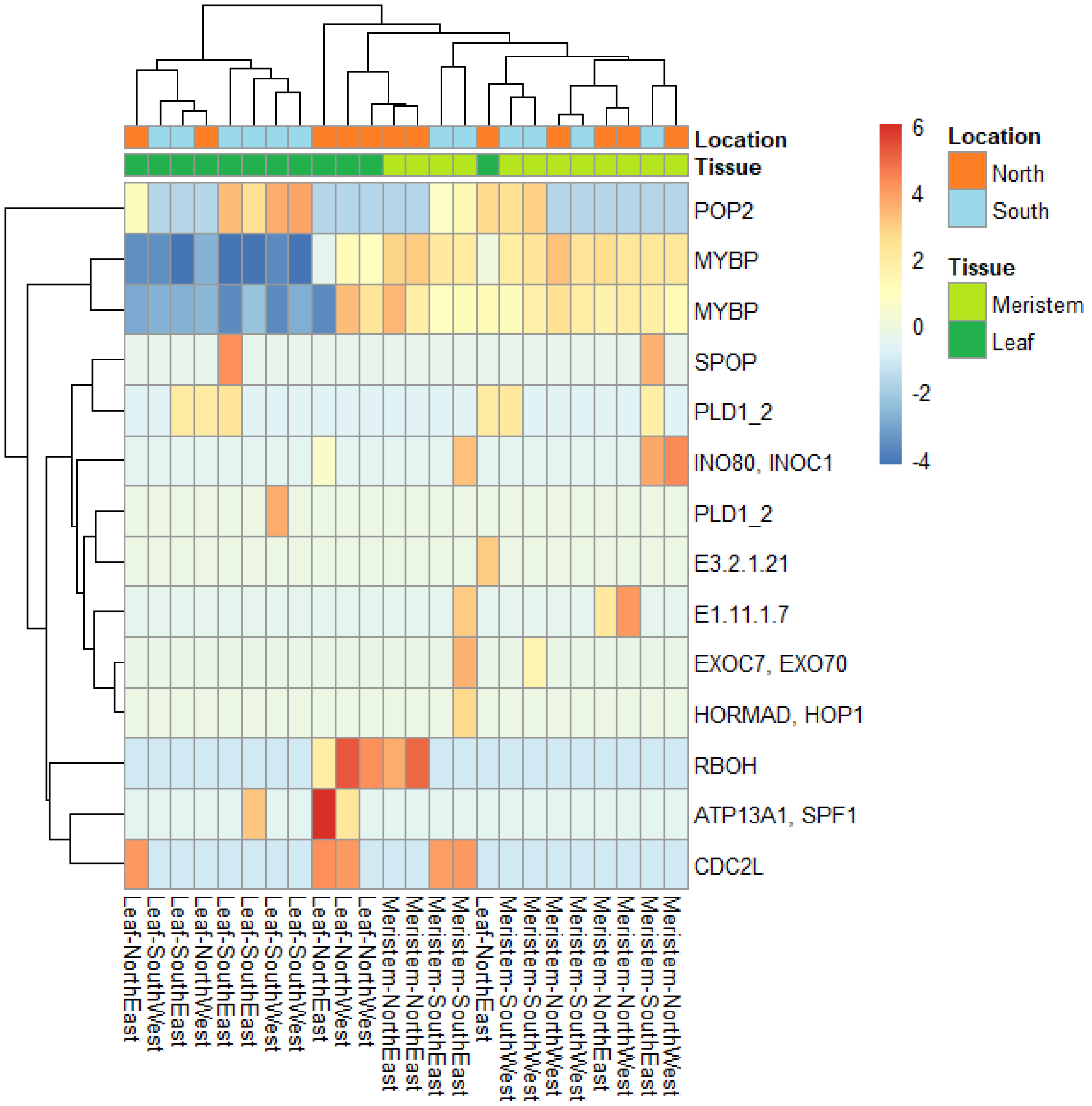

Fig. S11. Heatmap of all selection of important significantly enriched transcripts found from leaf and meristem tissue comparisons across salinities.

Heatmap showing the variance stabilising transformed counts of all transcripts with KEGG annotations (shown) from a selection of important significantly enriched biological processes across salinities. Tissue type and derived sampling locations represented according to the legends shown.

Biology Open • Supplementary information

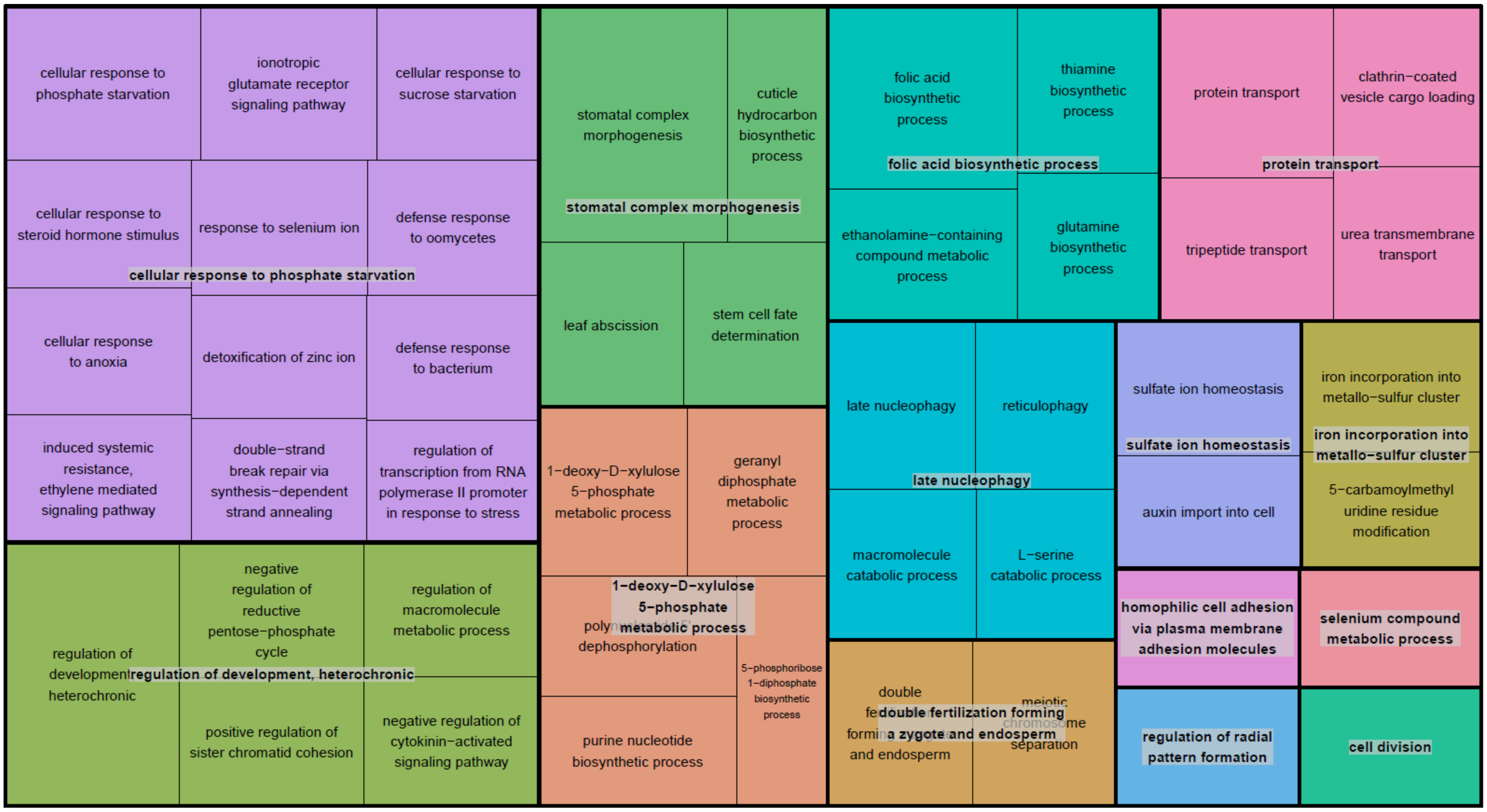

Fig. S12. GO term Treemap of upregulated transcripts from higher vs lower salinity comparison in leaf tissues.

Treemap of all significantly enriched "Biological Process" Gene Ontology terms ($P<0.05$) for all significantly upregulated ($q<0.05$, $LFC>|2|$) leaf transcripts from the leaf tissue comparison of higher vs lower salinity. All Gene Ontology terms were clustered into groups according to related terms to the cluster representative (group label) and coloured arbitrarily. Rectangle size reflects the absolute $\log_{10}$ value of the term's p-value.

Biology Open • Supplementary information

Table S8. Enriched GO term Treemap of upregulated transcripts from higher vs lower salinity comparison in leaf tissues.

Table of all significantly enriched "Biological Process" Gene Ontology terms (P<0.05) for all significantly upregulated (q<0.05, LFC>|2|) leaf transcripts from the leaf tissue comparison of higher vs lower salinity.

GO ID	GO Term	P-value
GO:0040034	regulation of development, heterochronic	0.0014
GO:0010103	stomatal complex morphogenesis	0.0017
GO:0046656	folic acid biosynthetic process	0.0068
GO:0016036	cellular response to phosphate starvation	0.0135
GO:0015031	protein transport	0.0137
GO:0035235	ionotropic glutamate receptor signaling pathway	0.0204
GO:0048366	leaf development	0.0245
GO:0044805	late nucleophagy	0.0278
GO:0043617	cellular response to sucrose starvation	0.0278
GO:0052863	1-deoxy-D-xylulose 5-phosphate metabolic process	0.0278
GO:0042939	tripeptide transport	0.0278
GO:0042439	ethanolamine-containing compound metabolic process	0.0278
GO:0010444	guard mother cell differentiation	0.0278
GO:0080153	negative regulation of reductive pentose-phosphate cycle	0.0278
GO:0055063	sulfate ion homeostasis	0.0278
GO:0009567	double fertilization forming a zygote and endosperm	0.0312
GO:0051307	meiotic chromosome separation	0.0331
GO:0071367	cellular response to brassinosteroid stimulus	0.0332
GO:0071383	cellular response to steroid hormone stimulus	0.0332
GO:0060866	leaf abscission	0.0333
GO:0018283	iron incorporation into metallo-sulfur cluster	0.0333
GO:0035652	clathrin-coated vesicle cargo loading	0.0333
GO:0098507	polynucleotide 5' dephosphorylation	0.0333
GO:0006723	cuticle hydrocarbon biosynthetic process	0.0333
GO:0009228	thiamine biosynthetic process	0.0333
GO:0071454	cellular response to anoxia	0.0333
GO:0045876	positive regulation of sister chromatid cohesion	0.0333

Biology Open • Supplementary information

GO:0015986	ATP synthesis coupled proton transport	0.0333
GO:0048867	stem cell fate determination	0.0333
GO:0061709	reticulophagy	0.0333
GO:0009866	induced systemic resistance, ethylene mediated signaling pathway	0.0333
GO:0033383	geranyl diphosphate metabolic process	0.0333
GO:0010269	response to selenium ion	0.0333
GO:0060255	regulation of macromolecule metabolic process	0.0345
GO:0002229	defense response to oomycetes	0.036
GO:0007130	synaptonemal complex assembly	0.0387
GO:0071918	urea transmembrane transport	0.0387
GO:0080037	negative regulation of cytokinin-activated signaling pathway	0.0387
GO:0009057	macromolecule catabolic process	0.0402
GO:0007156	homophilic cell adhesion via plasma membrane adhesion molecules	0.0442
GO:0010312	detoxification of zinc ion	0.0442
GO:0090213	regulation of radial pattern formation	0.0442
GO:0001887	selenium compound metabolic process	0.0442
GO:0045003	double-strand break repair via synthesis-dependent strand annealing	0.0442
GO:0042766	nucleosome mobilization	0.0442
GO:0006164	purine nucleotide biosynthetic process	0.0443
GO:0042742	defense response to bacterium	0.0448
GO:0051301	cell division	0.0456
GO:0043618	regulation of transcription from RNA polymerase II promoter in response to stress	0.0495
GO:0060919	auxin influx	0.0495
GO:0006565	L-serine catabolic process	0.0495
GO:0006542	glutamine biosynthetic process	0.0495
GO:0080178	5-carbamoylmethyl uridine residue modification	0.0495
GO:0006015	5-phosphoribose 1-diphosphate biosynthetic process	0.0495

Biology Open • Supplementary information

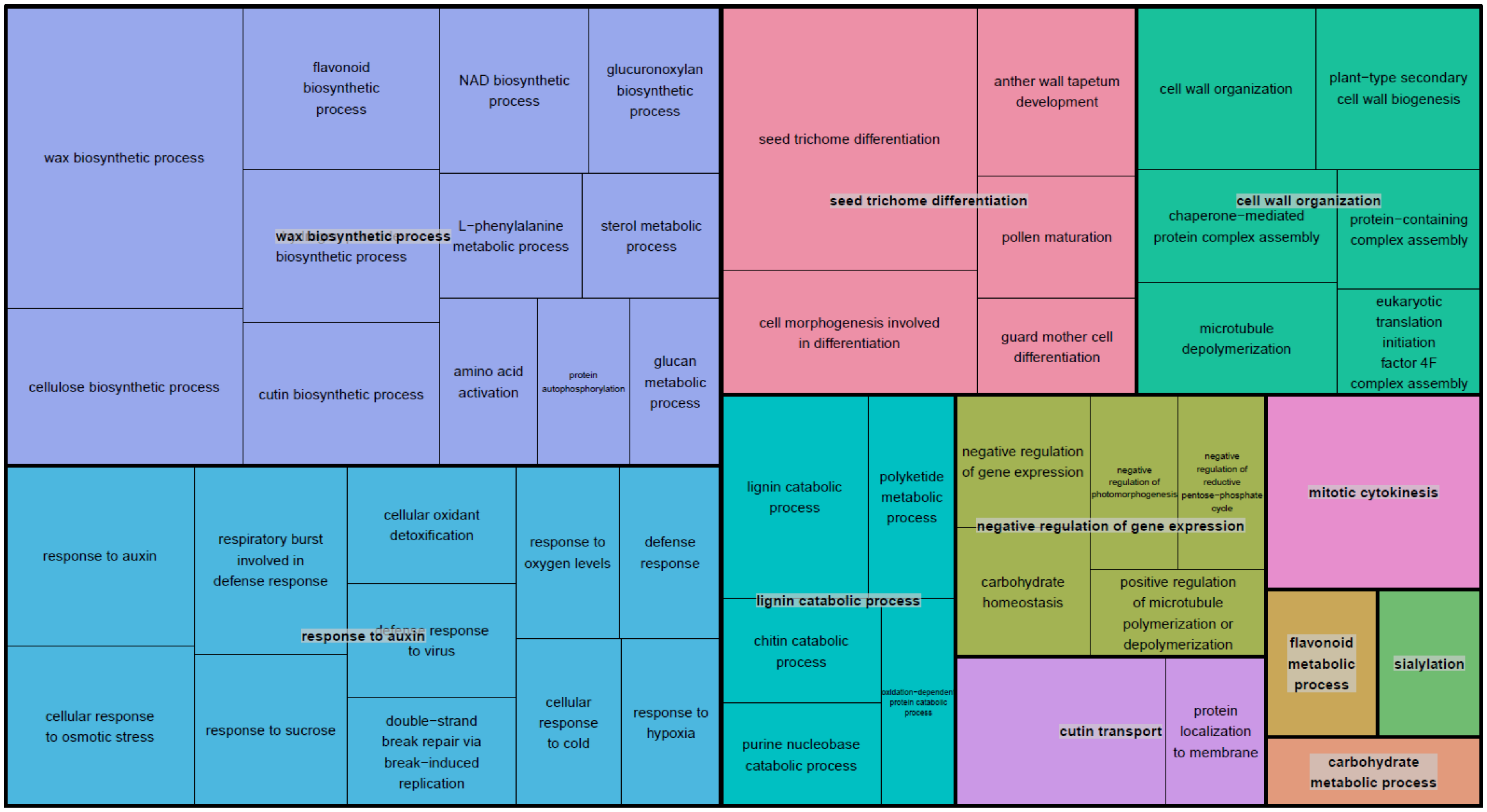

Fig. S13. GO term Treemap of downregulated transcripts from higher vs lower salinity comparison in leaf tissues.

Treemap of all significantly enriched "Biological Process" Gene Ontology terms (P<0.05) for all significantly downregulated (q<0.05, LFC>|2|) leaf transcripts from the leaf tissue comparison of higher vs lower salinity. All Gene Ontology terms were clustered into groups according to related terms to the cluster representative (group label) and coloured arbitrarily. Rectangle size reflects the absolute $\log_{10}$ value of the term's p-value.

Biology Open • Supplementary information

Table S9. Enriched GO terms of downregulated transcripts from higher vs lower salinity comparison in leaf tissues.

Table of all significantly enriched “Biological Process” Gene Ontology terms (P<0.05) for all significantly downregulated (q<0.05, LFC>|2|) leaf transcripts from the leaf tissue comparison of higher vs lower salinity.

GO ID	GO Term	P-value
GO:0010025	wax biosynthetic process	4.10E-07
GO:0090376	seed trichome differentiation	9.00E-07
GO:0000281	mitotic cytokinesis	0.00018
GO:0030244	cellulose biosynthetic process	0.00047
GO:0009733	response to auxin	0.00091
GO:0009813	flavonoid biosynthetic process	0.00137
GO:0000904	cell morphogenesis involved in differentiation	0.00144
GO:0080051	cutin transport	0.00157
GO:0071470	cellular response to osmotic stress	0.00206
GO:0050665	hydrogen peroxide biosynthetic process	0.00208
GO:0046274	lignin catabolic process	0.00208
GO:0071555	cell wall organization	0.0024
GO:0002679	respiratory burst involved in defense response	0.00266
GO:0010143	cutin biosynthetic process	0.00301
GO:0009834	plant-type secondary cell wall biogenesis	0.00395
GO:0048658	anther wall tapetum development	0.00402
GO:0009435	NAD biosynthetic process	0.00598
GO:0009739	response to gibberellin	0.00676
GO:0009744	response to sucrose	0.00858
GO:0042744	hydrogen peroxide catabolic process	0.00955
GO:0007019	microtubule depolymerization	0.0096
GO:0051131	chaperone-mediated protein complex assembly	0.0096
GO:0010417	glucuronoxylan biosynthetic process	0.01073
GO:0010588	cotyledon vascular tissue pattern formation	0.01318
GO:0016998	cell wall macromolecule catabolic process	0.01584
GO:0098869	cellular oxidant detoxification	0.01676
GO:0010152	pollen maturation	0.0187

Biology Open • Supplementary information

GO:0051607	defense response to virus	0.01887
GO:0000727	double-strand break repair via break-induced replication	0.02338
GO:0010629	negative regulation of gene expression	0.02502
GO:0070482	response to oxygen levels	0.02585
GO:0009832	plant-type cell wall biogenesis	0.02594
GO:0030638	polyketide metabolic process	0.02605
GO:0006558	L-phenylalanine metabolic process	0.02657
GO:0006952	defense response	0.0268
GO:0070417	cellular response to cold	0.02755
GO:0009735	response to cytokinin	0.02804
GO:0033500	carbohydrate homeostasis	0.02848
GO:0016125	sterol metabolic process	0.02873
GO:0065003	protein-containing complex assembly	0.02971
GO:0001666	response to hypoxia	0.03203
GO:0006032	chitin catabolic process	0.03211
GO:0006145	purine nucleobase catabolic process	0.03399
GO:0009812	flavonoid metabolic process	0.03405
GO:0043038	amino acid activation	0.03483
GO:0009826	unidimensional cell growth	0.03849
GO:0046777	protein autophosphorylation	0.0419
GO:0044042	glucan metabolic process	0.04268
GO:0010383	cell wall polysaccharide metabolic process	0.04303
GO:0097010	eukaryotic translation initiation factor 4F complex assembly	0.04319
GO:0010444	guard mother cell differentiation	0.04319
GO:0031112	positive regulation of microtubule polymerization or depolymerization	0.04319
GO:0080153	negative regulation of reductive pentose-phosphate cycle	0.04319
GO:0010100	negative regulation of photomorphogenesis	0.04319
GO:0080056	petal vascular tissue pattern formation	0.04319
GO:0080057	sepal vascular tissue pattern formation	0.04319
GO:0070407	oxidation-dependent protein catabolic process	0.04319
GO:0097503	sialylation	0.04319
GO:0052542	defense response by callose deposition	0.04609
GO:0072657	protein localization to membrane	0.04641
GO:0005975	carbohydrate metabolic process	0.04774

Biology Open • Supplementary information

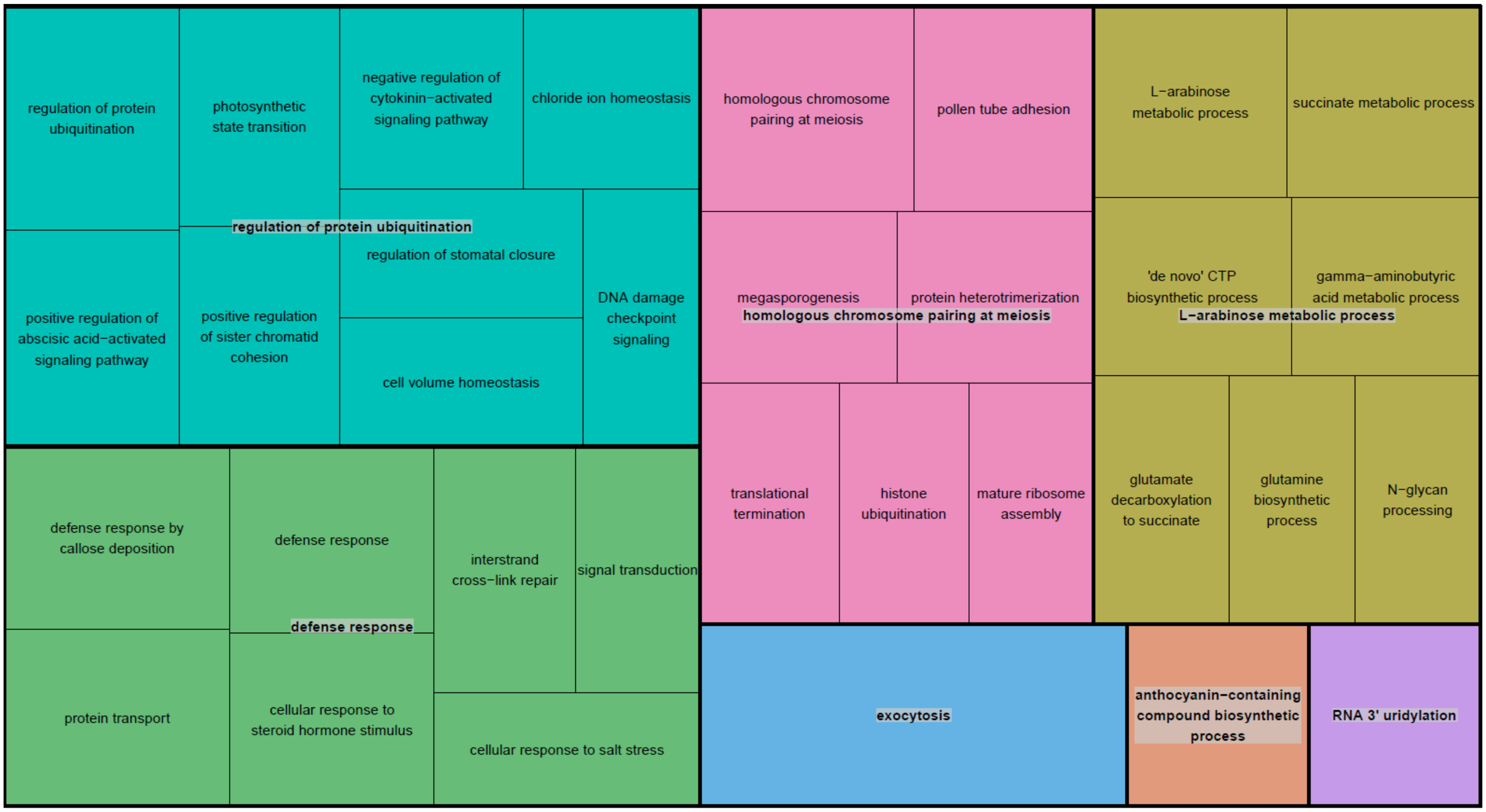

Fig S14. GO term Treemap of upregulated transcripts from higher vs lower salinity comparison in meristem tissues.

Treemap of all significantly enriched “Biological Process” Gene Ontology terms (P<0.05) for all significantly upregulated (q<0.05, LFC>|2|) meristem transcripts from the meristem tissue comparison of higher vs lower salinity. All Gene Ontology terms were clustered into groups according to related terms to the cluster representative (group label) and coloured arbitrarily. Rectangle size reflects the absolute $\log_{10}$ value of the term's p-value.

Biology Open • Supplementary information

Table S10. Enriched GO terms of upregulated transcripts from higher vs lower salinity comparison in meristem tissues.

Table of all significantly enriched “Biological Process” Gene Ontology terms (P<0.05) for all significantly upregulated (q<0.05, LFC>|2|) meristem transcripts from the meristem tissue comparison of higher vs lower salinity.

GO ID	GO Term	P-value
GO:0006887	exocytosis	0.0004
GO:0007129	homologous chromosome pairing at meiosis	0.0121
GO:0052542	defense response by callose deposition	0.0157
GO:0031396	regulation of protein ubiquitination	0.0193
GO:0015031	protein transport	0.021
GO:0006952	defense response	0.0216
GO:0009789	positive regulation of abscisic acid-activated signaling pathway	0.0223
GO:0046373	L-arabinose metabolic process	0.0243
GO:0006105	succinate metabolic process	0.0243
GO:0009865	pollen tube adhesion	0.0243
GO:0071367	cellular response to brassinosteroid stimulus	0.0289
GO:0071383	cellular response to steroid hormone stimulus	0.0289
GO:0044210	'de novo' CTP biosynthetic process	0.029
GO:0062055	photosynthetic state transition	0.029
GO:0045876	positive regulation of sister chromatid cohesion	0.029
GO:0036297	interstrand cross-link repair	0.0299
GO:0009554	megasporogenesis	0.0338
GO:0006540	glutamate decarboxylation to succinate	0.0338
GO:0080037	negative regulation of cytokinin-activated signaling pathway	0.0338
GO:0070208	protein heterotrimerization	0.0338
GO:0009448	gamma-aminobutyric acid metabolic process	0.0338
GO:0006415	translational termination	0.034
GO:0009718	anthocyanin-containing compound biosynthetic process	0.0361
GO:0055064	chloride ion homeostasis	0.0385

Biology Open • Supplementary information

GO:0090333	regulation of stomatal closure	0.0428
GO:0006542	glutamine biosynthetic process	0.0432
GO:0016574	histone ubiquitination	0.0432
GO:0071076	RNA 3' uridylation	0.0432
GO:0006491	N-glycan processing	0.0432
GO:0006884	cell volume homeostasis	0.0432
GO:0007165	signal transduction	0.0459
GO:0042256	mature ribosome assembly	0.0479
GO:0000077	DNA damage checkpoint	0.0487
GO:0071472	cellular response to salt stress	0.0487

Biology Open • Supplementary information

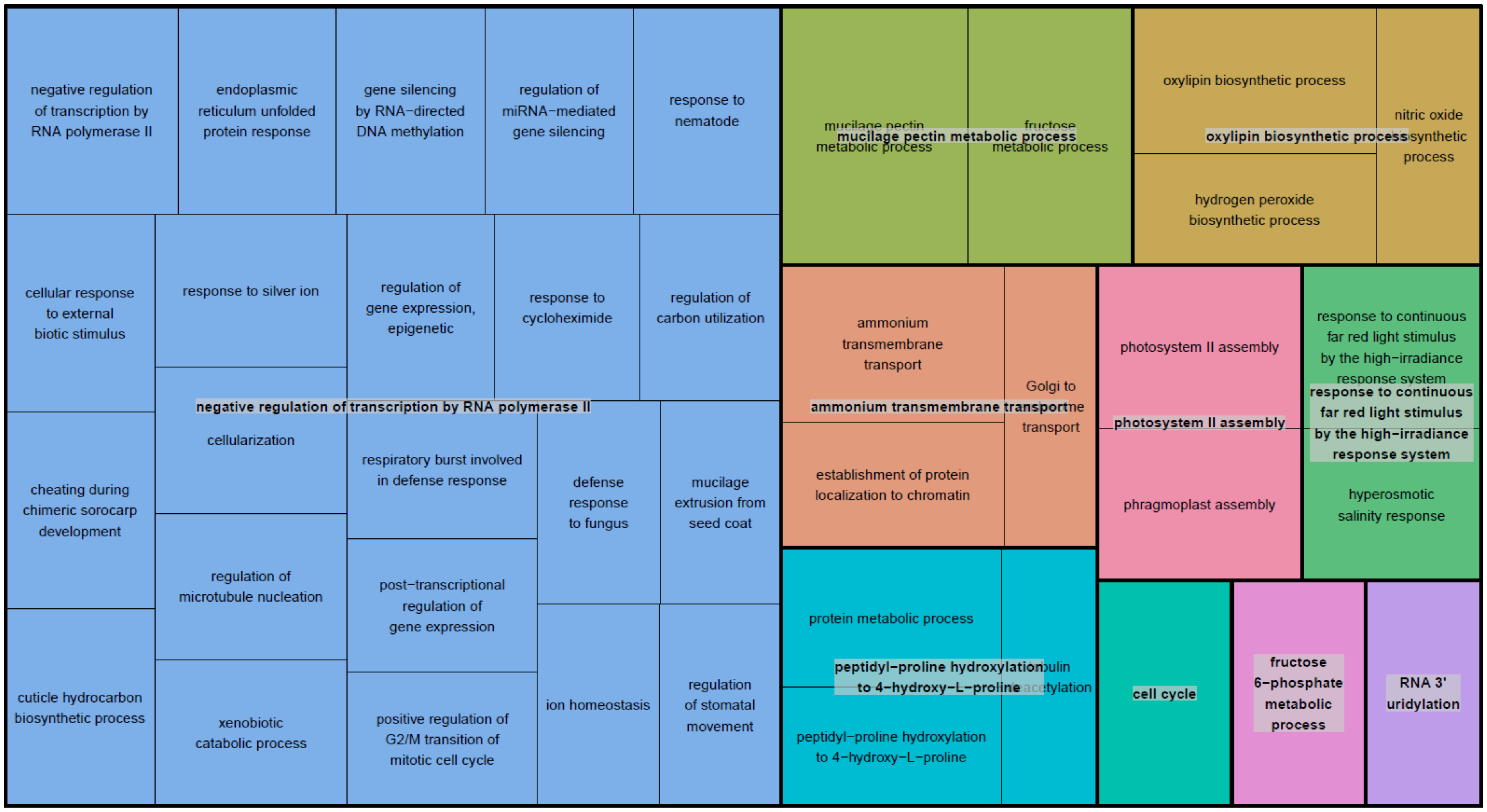

Fig. S15. GO term Treemap of downregulated transcripts from higher vs lower salinity comparison in meristem tissues. Treemap of all significantly enriched "Biological Process" Gene Ontology terms (P<0.05) for all significantly downregulated (q<0.05, LFC>|2|) meristem transcripts from the meristem tissue comparison of higher vs lower salinity. All Gene Ontology terms were clustered into groups according to related terms to the cluster representative (group label) and coloured arbitrarily. Rectangle size reflects the absolute $\log_{10}$ value of the term's p-value.

Table S11. Enriched GO terms of downregulated transcripts from higher vs lower salinity comparison in meristem tissues.

Table of all significantly enriched “Biological Process” Gene Ontology terms (P<0.05) for all significantly downregulated (q<0.05, LFC>|2|) meristem transcripts from the meristem tissue comparison of higher vs lower salinity.

GO ID	GO Term	P-value
GO:0048363	mucilage pectin metabolic process	0.0026
GO:0006000	fructose metabolic process	0.0052
GO:0031408	oxylipin biosynthetic process	0.012
GO:0000122	negative regulation of transcription by RNA polymerase II	0.012
GO:0015696	ammonium transport	0.0133
GO:0010207	photosystem II assembly	0.016
GO:0030968	endoplasmic reticulum unfolded protein response	0.0174
GO:0080188	gene silencing by RNA-directed DNA methylation	0.0222
GO:0000914	phragmoplast assembly	0.0222
GO:0060964	regulation of gene silencing by miRNA	0.0222
GO:0019538	protein metabolic process	0.0224
GO:0009624	response to nematode	0.0228
GO:0007049	cell cycle	0.0237
GO:0006002	fructose 6-phosphate metabolic process	0.0245
GO:0010272	response to silver ion	0.0266
GO:0010201	response to continuous far red light stimulus by the high-irradiance response system	0.0266
GO:0071217	cellular response to external biotic stimulus	0.0266
GO:0099139	cheating during chimeric sorocarp development	0.0266
GO:0006723	cuticle hydrocarbon biosynthetic process	0.0266
GO:0010968	regulation of microtubule nucleation	0.031
GO:0007349	cellularization	0.031
GO:0042178	xenobiotic catabolic process	0.031
GO:0071169	establishment of protein localization to chromatin	0.031
GO:0040029	regulation of gene expression, epigenetic	0.0343
GO:0006809	nitric oxide biosynthetic process	0.0353
GO:0046898	response to cycloheximide	0.0353

Biology Open • Supplementary information

GO:0050665	hydrogen peroxide biosynthetic process	0.0353
GO:0042538	hyperosmotic salinity response	0.0355
GO:0043609	regulation of carbon utilization	0.0396
GO:0018401	peptidyl-proline hydroxylation to 4-hydroxy-L-proline	0.0396
GO:0002679	respiratory burst involved in defense response	0.0396
GO:0071076	RNA 3' uridylation	0.0396
GO:0006895	Golgi to endosome transport	0.0396
GO:0010608	post-transcriptional regulation of gene expression	0.0437
GO:0010971	positive regulation of G2/M transition of mitotic cell cycle	0.0439
GO:0050832	defense response to fungus	0.0459
GO:0016458	gene silencing	0.0481
GO:0080001	mucilage extrusion from seed coat	0.0482
GO:0090042	tubulin deacetylation	0.0482
GO:0043447	alkane biosynthetic process	0.0482
GO:0050801	ion homeostasis	0.0484
GO:0010119	regulation of stomatal movement	0.0486
